# Generative adversarial network constrained multiple loss autoencoder: A deep learning‐based individual atrophy detection for Alzheimer's disease and mild cognitive impairment

**DOI:** 10.1002/hbm.26146

**Published:** 2022-11-17

**Authors:** Rong Shi, Can Sheng, Shichen Jin, Qi Zhang, Shuoyan Zhang, Liang Zhang, Changchang Ding, Luyao Wang, Lei Wang, Ying Han, Jiehui Jiang

**Affiliations:** ^1^ School of Information and Communication Engineering Shanghai University Shanghai China; ^2^ Department of Neurology Xuanwu Hospital of Capital Medical University Beijing China; ^3^ Key Laboratory of Biomedical Engineering of Hainan Province School of Biomedical Engineering, Hainan University Haikou China; ^4^ College of Computing and Informatics Drexel University Philadelphia Pennsylvania USA; ^5^ Center of Alzheimer's Disease Beijing Institute for Brain Disorders Beijing China; ^6^ National Clinical Research Center for Geriatric Disorders Beijing China; ^7^ Institute of Biomedical Engineering School of Life Science, Shanghai University Shanghai China

**Keywords:** Alzheimer's disease, GAN, magnetic resonance imaging, precision medicine

## Abstract

Exploring individual brain atrophy patterns is of great value in precision medicine for Alzheimer's disease (AD) and mild cognitive impairment (MCI). However, the current individual brain atrophy detection models are deficient. Here, we proposed a framework called generative adversarial network constrained multiple loss autoencoder (GANCMLAE) for precisely depicting individual atrophy patterns. The GANCMLAE model was trained using normal controls (NCs) from the Alzheimer's Disease Neuroimaging Initiative cohort, and the Xuanwu cohort was employed to validate the robustness of the model. The potential of the model for identifying different atrophy patterns of MCI subtypes was also assessed. Furthermore, the clinical application potential of the GANCMLAE model was investigated. The results showed that the model can achieve good image reconstruction performance on the structural similarity index measure (0.929 ± 0.003), peak signal‐to‐noise ratio (31.04 ± 0.09), and mean squared error (0.0014 ± 0.0001) with less latent loss in the Xuanwu cohort. The individual atrophy patterns extracted from this model are more precise in reflecting the clinical symptoms of MCI subtypes. The individual atrophy patterns exhibit a better discriminative power in identifying patients with AD and MCI from NCs than those of the *t*‐test model, with areas under the receiver operating characteristic curve of 0.867 (95%: 0.837–0.897) and 0.752 (95%: 0.71–0.790), respectively. Similar findings are also reported in the AD and MCI subgroups. In conclusion, the GANCMLAE model can serve as an effective tool for individualised atrophy detection.

## Graphical abstract

Our study presented a generative adversarial network constrained multiple loss autoencoder (GANCMLAE) model for the detection of individual atrophy patterns based on structural magnetic resonance imaging data. Experiments on two independent cohorts of participants showed that the residual maps from GANCMLAE model may serve as an effective tool to achieve precisely individualized atrophy detection and have potential for clinical applications.

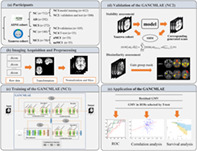

## 1 INTRODUCTION

The prevalence of Alzheimer's disease (AD) is projected to triple worldwide by 2050, imposing a heavy burden on patients, caregivers, and the social economy (C. Baur et al., 2020; Jia et al., 2018; Scheltens et al., 2021). Structural magnetic resonance imaging (MRI) is widely employed to reveal brain global and regional anatomical changes in AD because of its nonradiative characteristics (Evans et al., 2018; Lombardi et al., 2020; Xie et al., 2020). The current diagnostic criteria for AD recommend structural MRI to assist in the early detection of AD (McKhann et al., 2011).

Previous studies have reported that patients with AD exhibit certain general atrophy patterns. Medial temporal cortical atrophy, such as in the hippocampus and entorhinal cortex, is considered a common hallmark of AD (Devanand et al., 2007; M. Zhou et al., 2016). Using structural MRI, many studies have shown a significant grey matter (GM) volume reduction in the hippocampus, and those who ultimately converted to AD dementia also presented the decreased hippocampal volume in the early stages of AD (Devanand et al., 2007; Evans et al., 2018; Sheng et al., 2020; Whitwell et al., 2007; Xie et al., 2020). However, AD is a heterogeneous neurodegenerative disorder, accompanied by significant individual heterogeneity in brain atrophy patterns (Badhwar et al., 2020; Noh et al., 2014; Poulakis et al., 2018). Studies have indicated that AD has different atrophy subtypes. For instance, in addition to the predominant medial temporal atrophy, several AD patients exhibit parieto‐occipital atrophy, mild atrophy, and diffuse cortical atrophy patterns (Ten Kate et al., 2018). Moreover, these existing AD structural biomarkers can also be observed in other conditions, such as normal aging, frontotemporal lobe degeneration, and vascular dementia (Bastos‐Leite et al., 2007; Pleizier et al., 2012). Notably, the spatial distribution of brain atrophy on structural MRI is also highly heterogeneous in mild cognitive impairment (MCI), which is considered as a prodromal stage of AD (Nettiksimmons et al., 2014). Recent studies have reported different atrophy patterns between amnestic MCI (aMCI) and other MCI subtypes (Eliassen et al., 2015; Emmert et al., 2021; Sun et al., 2019). Emmert et al. found that the hippocampal volume is significantly lower in aMCI than in non‐aMCI (naMCI) (Emmert et al., 2021). Identifying the clinical subtypes of MCI remains challenging. Therefore, exploring the individual atrophy patterns of AD and MCI is crucial to achieve individualised diagnosis and is an important step towards precision medicine.

Currently, the conventional structural MRI‐based studies mainly involve manual partitioning of each image into a number of priority regions of interest or direct comparison of morphological differences at a whole‐brain level based on Student's *t* test of significance (Zhang et al., 2016). These studies typically reported differences between AD patients and NCs at a group level (Liu et al., 2018). However, group‐level atrophy pattern extraction approaches have relatively high data perturbations and low repeatability (Liu et al., 2018; Zhang et al., 2016). Moreover, accurately detecting AD using MRI is contingent on the signal‐to‐noise ratio (SNR) of the scan data, which is directly associated with instrument‐related parameters (X. Zhou et al., 2021). Owing to the limitations of conventional atrophy‐pattern extraction methods, a novel individual brain atrophy detection model is required to partially address the current issue (Logan et al., 2021).

Deep learning models are good choices. Early work on deep learning based on unsupervised anomaly detection approaches for brain MRI mainly relied on the classic autoencoder (AE) to model the normative distribution and find abnormalities (Atlason et al., 2019a; Christoph Baur et al., 2019). In particular, generative adversarial networks (GANs) are effective at generating tasks owing to their outstanding data learning and fitting capabilities. They can satisfactorily retain the desired information and facilitate MRI feature reconstruction using a generator and discriminator. For instance, Chong and Ho (2021) had used multiple GANs to separately learn the shape and texture of normal three‐dimensional (3D) brain MRI images for better generation. Kazemifar et al. (2019) achieved high dosimetry accuracy in synthetic computed tomography images generated from MRI data for focal brain radiation therapy using a GAN. GANs have made individual atrophy pattern extraction possible and helped detect abnormalities based on medical images. Guan et al. (2021) proposed an attention‐guided deep domain adaptation framework and applied it to automated brain disorder identification with multi‐site MRIs, which can also automatically identify discriminative regions in whole‐brain MRI images, and applied it to automated brain disorder identification with multi‐site MRI. Chen and Konukoglu (2018) also applied GANs for the detecting lesions in brain MRI images and determining the tumour location. The GANs showed great performance. Therefore, we hypothesised that GANs could improve the performance of the classic AE‐based framework in exploring individual atrophy patterns of patients with AD and MCI.

In the present study, we propose an optimised GAN‐based framework called the GANCMLAE model, which innovatively combines the GAN and AE, and further constrains the multiple losses to improve the identification of AD individual atrophy. The main purposes of this study are as follows: (1) to establish the GANCMLAE model, which is trained from NCs based on structural MRI data; (2) to validate the robustness of this GANCMLAE model in cross‐cultural cohorts comprising Chinese and American subjects; and (3) to investigate the clinical application potential of the model in detecting AD and MCI.

Our main contributions can be summarised as follows:

1. We developed the novel GANCMLAE model, characterised by the combination of a GAN and AE and constraining the multiple losses to improve the identification of individual brain atrophy in AD and MCI patients.
2. We proposed a two‐pronged strategy for validation: first, the robustness of this model was validated in two cohorts using the structural similarity index measure (SSIM), peak SNR (PSNR), and mean squared error (MSE) indices; second, the capability of this model in identifying different atrophy patterns of MCI subtypes was assessed and compared with that of the conventional group‐level *t*‐test method.
3. Extensive clinical experiments demonstrated that our model could identify individual atrophy patterns of AD and MCI, outperforming the group‐level *t*‐test model. Our framework has great potential for enhancing the clinical diagnosis of AD and MCI.

## 2 METHODS

### 2.1 Model

Because two‐dimensional (2D) networks are more widely used and the computational cost is smaller, this study designs the convolutional network based on 2D networks. Lesion detection is performed by first reconstructing and then detecting the lesion. In the first stage, the model is trained using health data such that the vector after dimensionality reduction can represent the feature distribution of the health data. In the second stage, the model is used to detect abnormal images. Because the model cannot learn abnormal features different from the health data, mapping errors will occur after reconstruction, which is regarded as lesions in the images. Based on this assumption, the GANCMLAE is designed.

**FIGURE 1:**
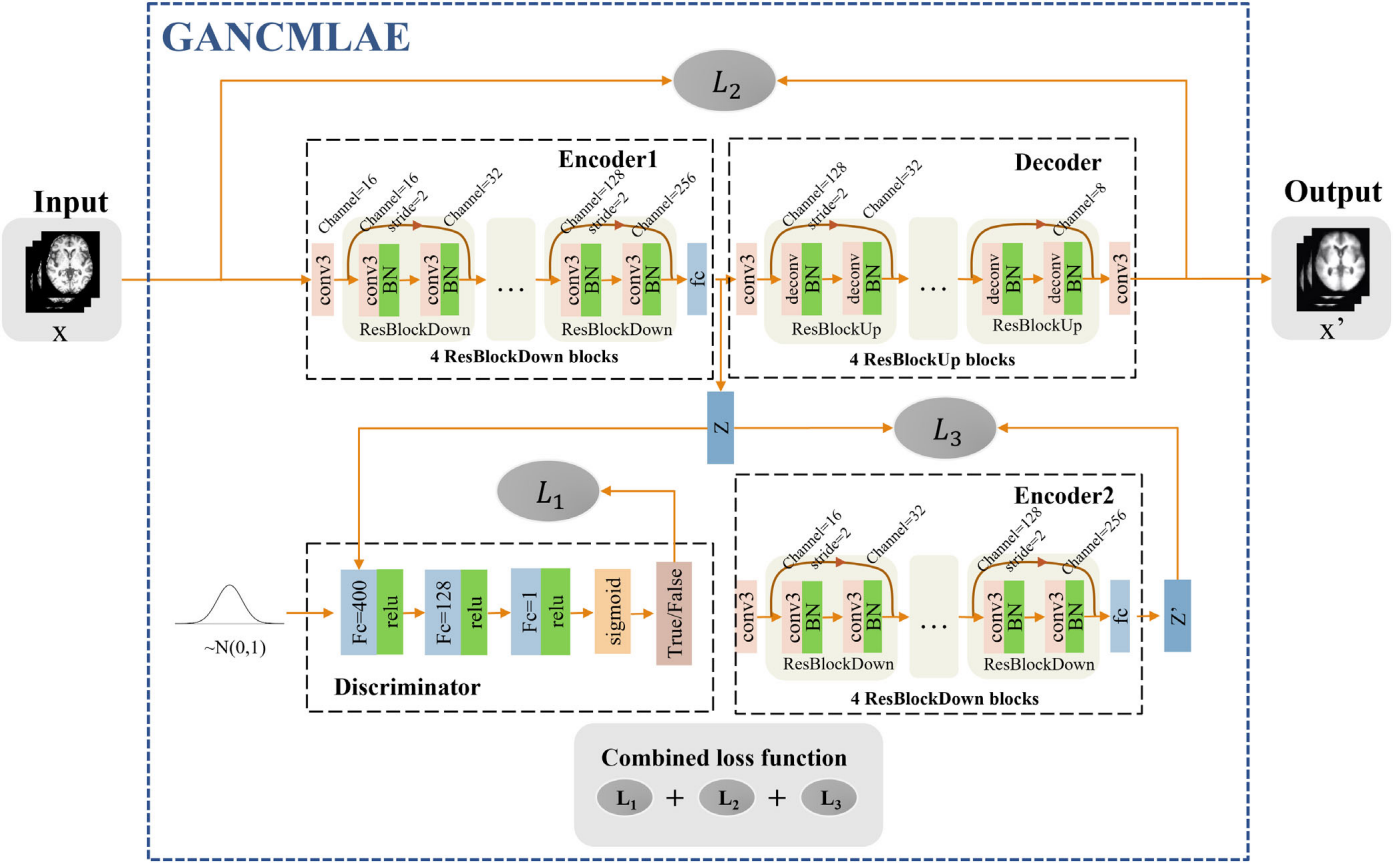
The overall architecture of the proposed generative adversarial network constrained multiple loss autoencoder (GANCMLAE). Encoder1, Decoder, Encoder2, Discriminator: the based structure; L1, L2, L3: the constituent elements of the combined loss; *x*: the input of the model; *z*: the generated latent vector of *x*, negative training data for the discriminator; *x*′: the reconstructed image; z′: the generated latent vector of *x*′. The vector sampled from the normal distribution: the positive training data for the discriminator

The overall architecture of the proposed GANCMLAE model is shown in Figure 1. The GANCMLAE model was previously trained using NC images. In this model, individual MRI images are used as inputs, and the residual images between the original input images and reconstructed images are the outputs. The above residual images are considered as the individual atrophy patterns, which are used by physicians to make clinical decisions.

Specifically, the basic framework consists of Encoder 1, Decoder, Encoder 2 and Discriminator. Encoder 1 and Decoder, functioning as the AE, are used for image reconstruction, whereas Discriminator and Encoder 2 constrain the intermediate processing by different losses. To better learn the global and local information of the images and simultaneously avoid the degradation problem, we implemented a multiple‐loss AE in contrast to the conventional GAN models. The details theories of these modules are shown as follows.

Encoder 1: Encoder 1 is designed to be composed of a convolutional layer and four residual blocks. In the first convolution, the number of filters is set to 16. In every subsequent downsampling residual block, the number of filters and output size are doubled so that we can obtain feature mapping with a size of 8 × 8 × 256. Feature selection is achieved through the full connection layer, during which a 1 × 128 vector is generated. In this way, the original 16,384 features are reduced to 128 features, and then these features are input into the model. Additionally, a batch normalisation (BN) layer is added following every convolutional layer to accelerate the convergence of the model. When a set of NC MRI scans $x_{h}\in R^{d*w*h}$, whose distribution is $P\left(x_{h}\right),$ is used for the model training, $\text{Encoder}1=Q\left(z|x_{h}\right)$ maps high‐dimensional data $x_{h}$ to low‐dimensional vector $z\in R^{z\_\dim}$, where $q\left(z\right)$ is the latent distribution. The distribution is calculated as follows:

(1) $$q\left(z\right)=\int Q\left(z|x_{h}\right)P\left(x_{h}\right)$$

Decoder: Similarly, the decoder consists of two convolutional layers and four upsampling residual blocks. The convolutional layers are used to maintain the required size of the mapping, and the deconvolutional layers followed by the BN layer can better reverse the encoder processing. Finally, the filter group sizes of the convolutional layer are 128, 128, 64, 64, 32, 32, 16, 16, 8, and 1. For all convolutions and deconvolutions, the kernel size is 3 × 3. The distribution is calculated as follows:

(2) $$P\left(x^{\prime }\right)=\int P\left(x_{h}|z\right)P\left(z\right)$$

where $x^{\prime }$ is the reconstruction of $x_{h}\;\mathrm{by}\;\text{the mapping Decoder}=P\left(x_{h}|z\right)$.

Encoder 2: Encoder 2 shares the same structure and parameters as Encoder 1. We map the reconstructed images to the output vector *z*′ in the same manner to prevent information loss or change caused by the decoder.

Discriminator: In classic AEs, there is no regularisation of the manifold structure z. In this model, we aim to generate a latent representation into a fixed distribution. Therefore, the discriminator imposes the aggregation posterior distribution *q*(*z*) to match an arbitrary prior distribution *P*(*z*) to realise regularisation. The role of the discriminative model is to determine whether a sample is from the generative model distribution $q\left(z\right)$ or the real data distribution *P*(*z*). Then, Encoder 1 generates a new sample from the input sample and makes the new sample satisfy the real distribution that we assumed to be Gaussian as much as possible. For the simple structure of the latent vector *z*, the discriminator in our study has three fully connected layers instead of a convolutional layer: one with 256 units, one with 128 units, and one with 1 unit. Every fully connected layer is followed by a LeakyReLU operation. After the last sigmoid activation, the score indicates the performance of the input.

Loss function: Similar to general GAN models, the loss of the basic GANCMLAE is limited to identifying hidden layer distributions, but the quality and feature retention of image restoration cannot be guaranteed. Thus, our model combines several losses for training. The loss that reaches stabilisation is expressed as:

(3) $$L_{\text{balance}}=\min\left(L_{1}\right)+\text{μmin}\left(L_{2}\right)+\text{γmin}\left(L_{3}\right)$$

$L_{1}$: The model combines several losses for training. Fundamentally, the generator simulates the real distribution by optimising parameters to trick the discriminator, and the discriminator trains the parameters to identify the input. The system finally reaches the state of “Nash balance” in the process of alternative optimisation. The original target, the GANCMLAE, is represented as

(4) $$\begin{array}{rcl} \min_{G}\max_{G}V\left(D,G\right) & = & E_{z\sim P\left(z\right)}\left[\log D\left(z\right)\right]+E_{x\sim P\left(x_{h}\right)}\left[\log\left(1-D\left(\mathrm{Enc}\left(x\right)\right)\right)\right]\\ & = & E_{z\sim P\left(z\right)}\left[\log D\left(z\right)\right]+E_{z\sim q\left(z\right)}\left[\log\left(1-D\left(z\right)\right)\right] \end{array}$$

After derivation, we found that the loss is essentially JS dispersion. Compared to the KL dispersion applied in the variation encoder (VAE), JS dispersion solves the problem of asymmetry. However, there are still serious defects when the two distributions do not overlap, which results in a gradient of zero, which is fatal in model training. Thus, the Wasserstein distance was used for further optimisation. The loss function was expressed as follows:

(5) $$L_{1}=E_{z\sim q\left(z\right)}D\left(z\right)-E_{z\sim P\left(z\right)}D\left(z\right)+\lambda E_{z\sim P\left(\text{penalty}\right)}\left[\max\left(0,\left|\left|\nabla_{z}D\left(x\right)\right|\right|-1\right)\right]$$

where $P\left(\text{penalty}\right)$ is the intermediate distribution of the two distributions, and λ is 10 by default.

$L_{2}$: In addition, to better preserve the image details during reconstruction, we combined the adversarial loss with pixel‐wise loss, in which reconstruction losses are represented as the least absolute error:

(6) $$L_{2}=|x-x^{\prime }|$$

$L_{3}$: Furthermore, enhancing the consistency of the latent vector of the original input and that of its reconstructed image was proved to be beneficial for anomaly detection and for ease of implementation. We recoded the reconstructed image $x^{\prime }$ in a compilation to obtain its latent vector $z^{\prime }$. Then, to improve the consistency of the potential representation, we modified the original latent vector *z* by adding the regular term $L_{3}={\left|z-z\prime \right|}^{2}$ to the total loss.

### 2.2 Overall study design

**FIGURE 2:**
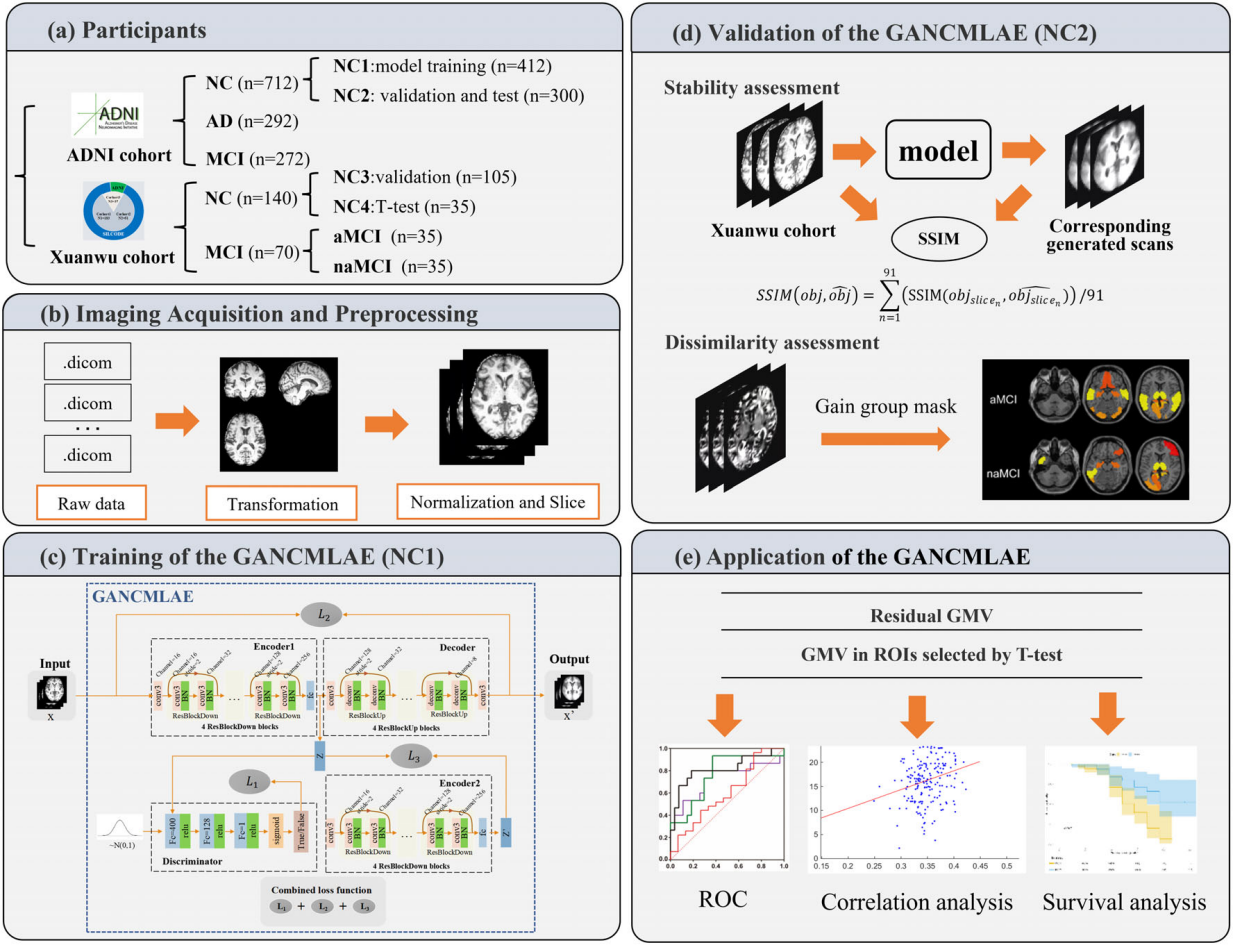
The comprehensive workflow of the generative adversarial network constrained multiple loss autoencoder (GANCMLAE) model. (a) Two cohorts were enrolled. Cohort A was from the ADNI and cohort B were from the SILCODE project. (b) The procedures of structural magnetic resonance imaging (MRI) processing for model training. (c) Train the model with normal controls (NCs) and then reconstruct the input data including NC, Alzheimer's disease (AD), or mild cognitive impairment (MCI). Residual scans can be gained from the input and output. (d) Evaluate the performance of the model from two aspects: a. structural similarity index measure (SSIM) of original NC scans and generated ones from the cohort B are used to prove the robustness; and (e) application of the GANCMLAE model in AD and MCI

In this study, the MRI data were obtained from the AD Neuroimaging Initiative (ADNI) and the Xuanwu cohorts. After image preprocessing, the data of NC individuals in the ADNI cohort were used to train the GANCMLAE model. To validate the robustness and effectiveness of the model, we used a two‐pronged approach. First, the SSIM, PSNR, and MSE indices were employed to validate the image reconstruction robustness using NC individuals from both the ADNI and Xuanwu cohorts. Second, the clinical application potential of the model was investigated to verify its capability for identifying different subtypes of MCI and AD from NCs. Notably, the group‐level *t*‐test model was chosen as the comparison model because it is most frequently used for atrophy detection in previous studies (Bakkour et al., 2009; Devanand et al., 2007; Dickerson et al., 2009; Lombardi et al., 2020; Popuri et al., 2020; Whitwell et al., 2007). The overall flow of this study included the following: (1) participant recruitment from the ADNI cohort and the Xuanwu cohorts; (2) the GANCMLAE model training using structural MRI images of NC individuals; (3) reconstruction of the input data including NC, AD, and MCI and acquisition of residual scans; (4) validation of the GANCMLAE model; and (5) application of the GANCMLAE model in AD and MCI. The detailed workflow of this study is illustrated in Figure 2.

### 2.3 Participants

The participants were selected from two independent cohorts: ADNI cohort (cohort A) from the ADNI database (http://adni.loni.usc.edu/) and Xuanwu cohort (cohort B) from the Sino Longitudinal Study on Cognitive Decline. In cohort A, 712 NC, 292 AD, and 309 MCI samples were collected. Among them, 412 NC individuals (NC1 group) were used for GANCMLAE model training, and 300 NC (NC2 group) individuals were used for internal validation. Cohort B comprised 140 NC, 35 aMCI, and 35 naMCI individuals. The SSIM index was calculated to validate the robustness of the trained GANCMLAE model using 105 NC individuals (NC3 group) from cohort B as the external validation data set. To further assess the ability of our model to detect individual atrophy, characteristic atrophy masks associated with aMCI and naMCI were extracted separately from the remaining 35 NC individuals (NC4 group) from cohort B as a reference. All participants in cohort A underwent the following neuropsychological examinations: Clinical Dementia Rating‐Sum of Boxes (CDR‐SB) and Mini‐Mental State Examination (MMSE). For the participants in the cohort B, the Auditory Verbal Learning Test (AVLT) and Montreal Cognitive Assessment (MoCA) were performed. MMSE was not the regular neuropsychological assessment in the recruitment. The demographic information (sex, age, education, APOE) and T1‐weighted structural MRI data for all participants were collected.

In cohort B, MCI was defined in accordance with the criteria proposed by Jak and Bondi (Bondi et al., 2014). Participants were diagnosed with MCI if they met any one of the following three criteria and failed to meet the criteria for dementia: (1) having impaired scores (defined as >1.0 SD below the age/education‐corrected normative means) on both measures in at least one cognitive domain (memory, language, or speed/executive function); (2) having impaired scores in each of the three cognitive domains (memory, language, or speed/executive function); and (3) Functional Activities Questionnaire ≥9. Individuals with memory complaints and objective memory decline were considered as aMCI patients (Sheng et al., 2020), and those without significant deficits in the memory domain were regarded as naMCI patients. The diagnosis of AD dementia was based on the Diagnostic and Statistical Manual of Mental Disorders (fifth edition) and guidelines for dementia due to AD issued by the NIA‐AA workgroup (McKhann et al., 2011).

The institutional review board of ADNI reviewed and approved the ADNI data collection protocol. The research activities involving the Xuanwu cohort were approved by the Medical Research Ethics Committee and Institutional Review Board of Xuanwu Hospital in Capital Medical University (ID: [2017]046). All participants provided written informed consent before participating in the study.

### 2.4 Image acquisition and preprocessing

#### 2.4.1 Image acquisition

The structural images of participants in cohort A were obtained from the ADNI database. Detailed information regarding the acquisition protocol is publicly available on the LONI website. The structural MRI data of participants from cohort B were acquired using a 3.0 T MRI scanner (Magnetom Sonata; Siemens Healthineers AG, Erlangen, Germany) or an integrated simultaneous 3.0 T time‐of‐flight PET/MR (SIGNA; GE Healthcare, Chicago, IL). The structural MRI images (Siemens scanner) were obtained with a magnetisation‐prepared rapid gradient echo sequence: repetition time (TR) = 1900 ms, echo time (TE) = 2.2 ms, and number of slices = 176. The parameters for the structural images (GE scanner) were as follows: FOV = 256 × 256 mm^2^, matrix = 256 × 256, slice thickness = 1 mm, gap = 0, number of slices = 192, TR = 6.9 ms, TE = 2.98 ms, inversion time (TI) = 450 ms, flip angle = 12°, and voxel size = 1 × 1 × 1 mm^3^.

#### 2.4.2 Image preprocessing

The image preprocessing included the following steps: (1) The Nicom format of the data was converted to the Neuroimaging Informatics Technology Initiative (NifTI) format using the DCM2NII (https://people.cas.sc.edu/rorden/mricron/dcm2nii.html) tool. (2) To adapt and speed up the training of the deep learning model, the images were normalised to −1 to 1, and then sliced from the axial direction into 91 single‐channel images with a size of 91 × 109. We cropped and resampled the slices to 128 × 128 using linear interpolation as model inputs and normalised them. (3) In the evaluation and statistics stages, the GM volume and the total intracranial volume (TIV) were used as measurement indicators. Therefore, we used cat12 to segment the GM image and calculate the corresponding TIV. The formula for normalising the image to −1 to 1 is as follows:

(7) $$x=\frac{x-\text{mean}\left(x\right)}{\max\left(x\right)-\min\left(x\right)}\;$$

where *x* represents the image, and mean(*x*), max(*x*), and min(*x*) represent the average, maximum, and minimum voxel values, respectively.

### 2.5 Training of GANCMLAE

The GANCMLAE model is an end‐to‐end network. We used the two time‐scale update rule strategy to adjust the learning rate of the generator and discriminator, and it was not strictly alternate training as in the original literature, but the discriminator was trained twice for each training. We then set the learning rate as $2e^{-4}$ for both the generator and discriminator. We assessed the training situation by observing the gradient change of each loss using the visualisation tool TensorBoard.

To visually display the generated images and their corresponding residual images, we selected two NC individuals (NC (1): age = 66.4, MMSE = 29; NC (2): age = 63.1, MMSE = 28) and two patients with AD (AD (1): age = 72.7, MMSE = 10; AD (2): age = 65.9, MMSE = 8) from the ADNI cohort. The NC or AD individuals have similar clinical information and cognitive performance.

### 2.6 Validation of GANCMLAE

#### 2.6.1 Evaluation metrics

In our model, the MRI images of each normal group were restored after dimension reduction. However, because the loss was based on a combination of customised indicators, it had no practical comparison significance. Therefore, this study introduced the SSIM, PSNR, and MSE as the measurement indices.

The SSIM is a measure of the similarity between two images and ranges from −1 to 1. When two images are identical, the SSIM is 1. The SSIM is defined as follows (Equation (8)):

(8) $$\text{SSIM}\left(x,y\right)=\frac{\left(2\mu_{x}\mu_{y}+c_{1}\right)\left(2\sigma_{\mathrm{xy}}+c_{2}\right)}{\left(\mu_{x}^{2}+\mu_{y}^{2}+c_{1}\right)\left(\sigma_{x}^{2}+\sigma_{y}^{2}+c_{2}\right)}$$

where $\mu_{x}\;\text{and}\;\mu_{y}$ are the means of samples *x* and *y*, respectively; $\sigma_{x}^{2}\;\text{and}\;\sigma_{y}^{2}$ are the variances of samples *x* and *y*, respectively; $\sigma_{\mathrm{xy}}$ is the covariance of samples *x* and *y*; and $c_{1}\;\text{and}\;c_{2}$ are two constants determined by the range of pixel values. Given two images *I* and *K* with a size of m × n, the calculation formulas for MSE and PSNR are given by Equations 9 and 10, respectively. ${\mathrm{MAX}}_{I}$ is the maximum pixel value of the image.

(9) $$\mathrm{MSE}=\frac{1}{\mathrm{mn}}\sum_{i=0}^{m-1}\sum_{j=0}^{n-1}{\left\|I\left(i,j\right)-K\left(i,j\right)\right\|}^{2}$$

(10) $$\text{PSNR}=10\log\left(\frac{{\mathrm{MAX}}_{I}^{2}}{\mathrm{MSE}}\right)=20\log\left(\frac{{\mathrm{MAX}}_{I}}{\sqrt{\mathrm{MSE}}}\right)$$

In this study, all values were calculated for both internal and external validation data sets from the ADNI and Xuanwu cohorts. Because the model and metrics are all for 2D images, the index of each subject was obtained by averaging all slices of that individual.

#### 2.6.2 Performance comparison

We compared the performance of the following state‐of‐the‐art baselines to verify the superiority of the GANCMLAE model for unsupervised reconstruction. The hyperparameter selection of the models was based on previous studies. The SSIM, PSNR, and MSE values were used to evaluate the performance of the reconstruction results for NC2.

1. AEs (Atlason et al., 2019): Owing to their ability to learn nonlinear transformation of data from a low‐dimensional manifold, AEs have been widely used for cluster‐based anomaly detection.
2. VAE (Hou et al., 2017): The VAE constrains the latent space by leveraging the encoder and decoder networks of AEs to parameterise a latent distribution.
3. FAnoGAN (Schlegl et al., 2019): It connects and improves on the GAN and introduces an efficient way of replacing the costly iterative restoration method with a single forward pass through the network
4. DC‐CNN reconstruction (Schlemper et al., 2018): It is a deep cascade of convolutional neural network for reconstructing MRI images from undersampled data. Different from AE‐based models it can be used in performance comparison from another dimension.
5. AAE (Makhzani et al., 2015): It leverages an adversarial network as a proxy metric to minimise the discrepancy between the learned distribution and the prior one.

In addition, we also performed ablation experiments and compared our model with different loss functions including L1 + L2, L1 + L3, and L2 + L3.

#### 2.6.3 Dissimilarity assessment

To assess the dissimilarity of our proposed model, we captured and compared the individual atrophy patterns from different MCI subtypes. We selected 35 aMCI and 35 naMCI participants from the Xuanwu cohort. For each subtype, the corresponding individual residual map was obtained using the GANCMLAE model and the corresponding 3D image was reconstructed from 2D slices. To avoid noise and deviation, pixels with values greater than 0.03 were considered as effective atrophy with the threshold that noise outside the MNI region can just be removed. Based on Bertrand Thirion's procedure, we calculated the frequency of each pixel to identify the atrophic region and pixels with frequencies exceeding 60% were considered as residual masks of the subtypes. Simultaneously, 35 NC individuals (NC4 group) were used as a reference to conduct a t‐test with two subtypes. Using the GANCMLAE and *t*‐test, respective inter‐group masks were obtained by cluster processing with a size of 50 as the threshold. Both inter‐group masks of the GANCMLAE and *t*‐test methods were further compared.

### 2.7 Application of GANCMLAE

#### 2.7.1 Receiver operating characteristic analysis and classification with classical deep learning models

To compare the discriminative power of the individual atrophy patterns from the GANCMLAE model in identifying patients with AD and MCI from NCs with that of the *t*‐test model, the receiver operating characteristic (ROC) curve and area under the ROC curve (AUC) were calculated. According to the cut‐off of Aβ42 in the cerebrospinal fluid (CSF) proposed by Hu et al. (2019), AD+ (MCI+) patients were defined as <813 pg/ml for CSF Aβ42. In the subgroup analysis, patients with AD and MCI in the ADNI cohort were classified as amyloid‐positive AD/MCI (AD+, *n* = 73; MCI+, *n* = 45) and amyloid‐negative AD/MCI (AD−, *n* = 14; MCI−, *n* = 21). We further compared the discriminative power of the two models in distinguishing AD+ (MCI+) patients and AD− (MCI−) from the controls (NC2 group). In addition, we compared the discriminative powers of the GANCMLAE model and the *t*‐test models in identifying patients with aMCI and naMCI from the controls (NC4 group) in the Xuanwu cohort.

To further verify the validity of the residual maps, we applied them as inputs during the deep learning classification between AD and NC, and MCI and NC in the ADNI cohort. The performance values with residual maps, original images and original images supervised by *t*‐test masks were compared. Given that most classification models are based on 2D networks, in our study, the raw and residual structural MRI images were reduced to 224 × 224 after slicing and tiling. These images were then piled up and converted into three‐channel images. To eliminate the contingency factor, we employed four classic models for this task: LeNet, AlexNet, ResNet18, and ResNet34. A 10‐fold cross‐validation was performed before feature selection. The data set was randomly divided into 10 parts, with nine‐tenths of the data allocated for the training set and the rest for the validation set. The accuracy, sensitivity, specificity, AUC, F‐score, and Matthews correlation coefficient (MCC) were, respectively, calculated for the test set and validation sets.

#### 2.7.2 Correlation between the GANCMLAE model and cognitive function in AD and MCI

All slices were restored to a 3D format and the relative GM volume of the original and reconstructed subjects were calculated. The relationships of the individual atrophy patterns (the difference in the relative GM volume before and after reconstruction) from the GANCMLAE model and the general atrophy patterns from the *t*‐test model were separately assessed by partial correlation analysis with the global cognitive function (MMSE) and severity of cognition (CDR‐SB) in AD and MCI patients, using for age, sex, and years of education as the covariates.

In the subgroup analysis, using partial correlation analysis, the relationships of the two models with MMSE and CDR‐SB were also evaluated using age, sex, and years of education as covariates. We also evaluated the correlation between the two models and cognitive performance in APOE ε4 carriers (*n* = 134) and APOE ε4 noncarriers (*n* = 151), and further calculated the interaction effects of APOE ε4 and the models was further calculated.

#### 2.7.3 Survival analysis

Survival analysis was performed using the Kaplan–Meier method, and any differences in survival were evaluated using a log‐rank test. A total of 219 MCI patients from the ADNI cohort had longitudinal data (mean follow‐up period: 32.14 months), and MCI patients were converted into dementia (conversion rate: 18.72%). To assess the effectiveness of the individual atrophy patterns from the GANCMLAE model in predicting the conversion risk of MCI to dementia, we compared the survival probability between the standardised GANCMLAE residual score >0 group and the standardised GANCMLAE residual score <0 group. Hazard ratios (HRs) indicated the risk of conversion to dementia between the two groups. The *p*‐value was calculated using the log‐rank test. The standardised values (z‐scores) of the individual atrophy patterns from the GANCMLAE model were calculated using the following formula:

(11) $$Z\;\text{score}=\frac{\text{Individual}\;\mathrm{GAN}\;\text{residual score}-M_{1}}{{\mathrm{SD}}_{1}}$$

where *M*_1_ denotes the mean GANCMLAE residual score, and SD_1_ is the standard deviation of the GANCMLAE residual score.

### 2.8 Statistics and analysis

The Shapiro–Wilk test was used to confirm data normality. Demographic information and neuropsychological assessments were compared using the two‐sample *t* test, Kruskal–Wallis test, or Pearson's chi‐squared test as appropriate. Losses and indicators in model training, such as SSIM, were realised by the method in the core open source library TensorFlow and visualised using TensorBoard. The statistical parametric mapping (SPM8, https://www.fil.ion.ucl.ac.uk/spm/software/spm8) and DPABI (http://rfmri.org/dpabi) in MATLAB, and GraphPad Prism v9.0 were used to plot and visualise all statistical data in this study. The statistical significance was set at *p* < .05.

## 3 RESULTS

### 3.1 Demographic information and neuropsychological assessments

Table 1 presents the demographic and clinical details of all participants at the baseline. There are significant differences in age and years of education between NC1 (training data set in the ADNI cohort) and NC3 (validation data set for SSIM in the Xuanwu cohort) (*p* < .001), whereas no differences in sex and APOE ε4 carrier are observed. For the application data set from the ADNI cohort, significant differences in years of education, MMSE, CDR‐SB, and APOE ε4 carriers are observed between the NC1 and AD groups (*p* < .001), while the MCI group exhibits differences in sex, MMSE, CDR‐SB, and APOE ε4 carrier compared with the NC1 group (sex: *p* = .003; APOE ε4 carrier: *p* = .003; MMSE and CDR‐SB: *p* < .001). As indicated in the validation data set for dissimilarity, aMCI patients show significant differences in educational years, AVLT‐long delayed recall, AVLT‐recognition, and MoCA‐B compared with NC3 individuals (education: *p* = .001; AVLT‐long delayed recall, AVLT‐recognition and MoCA‐B: *p* < .001), whereas there are significant differences in educational level, AVLT‐long delayed recall and MoCA‐B between the naMCI and NC3 groups (education: *p* = .002; AVLT‐long delayed recall and MoCA‐B: *p* < .001).

**TABLE 1:** Demographic information and neuropsychological assessments

	ADNI cohort				Xuanwu cohort			
	Training data set	Application data set			Validation data set for SSIM	Validation data set for dissimilarity		
	NC1	AD	MCI	NC2	NC3	aMCI	naMCI	NC4
*N*	412	292	309	300	105	35	35	35
Age (years)	72.47 ± 6.06	72.96 ± 6.92	73.40 ± 7.36	71.79 ± 5.46	65.61 ± 5.53***	66.97 ± 7.63	64.89 ± 7.51	65.46 ± 4.94
Sex (F/M)	228/184	150/142	137/172^&&^	171/129	69/36	19/16	24/11	20/15
Education (years)	16.55 ± 2.52	15.35 ± 2.86^###^	16.17 ± 2.71	16.43 ± 2.38	12.29 ± 3.12***	10.11 ± 3.76^§§^	10.34 ± 3.31^††^	12.00 ± 2.68
MMSE	29.10 ± 1.10	21.15 ± 4.49^###^	26.13 ± 3.85^&&&^	28.83 ± 1.61	/	/	/	/
CDR‐SB	0.06 ± 0.21	5.44 ± 2.27^###^	2.68 ± 2.36^&&&^	0.25 ± 1.07	/	/	/	/
AVLT‐(long) D	/	/	/	/	8.22 ± 2.56	2.46 ± 2.10^§§§^	4.69 ± 2.30^†††^	7.74 ± 2.07
AVLT‐Recognition	/	/	/	/	20.47 ± 4.61	7.53 ± 4.77^§§§^	19.86 ± 3.01	22.83 ± 1.13
MoCA‐B	/	/	/	/	25.63 ± 3.04	18.6 ± 4.27^§§§^	20.77 ± 3.99^†††^	26.46 ± 2.03
APOE ε4	124 (30.09%)	134 (45.89%)^###^	126 (40.78%)^&&^	100 (33.33%)	32 (30.48%%)	15 (42.86%)	13 (37.14%)	7 (20.00%)
Aβ42 in CSF (Aβ+/Aβ−)	24/53	73/14	45/21	24/12	/	/	/	/
Follow‐up period for MCI (months)	/	/	32.14 ± 29.54	/	/	/	/	/
Converter rate of MCI	/	/	41 (18.72%)	/	/	/	/	/

Abbreviations: AD, Alzheimer's disease; aMCI, amnestic MCI; APOE, apolipoprotein E; AVLT, Auditory Verbal Learning Test; Aβ, amyloid β; CDR‐SB, Clinical Dementia Rating‐Sum of Boxes; CSF, cerebral spinal fluid; MCI, mild cognitive impairment; MMSE, Mini‐Mental State Examination; MoCA, Montreal Cognitive Assessment; naMCI, non‐aMCI; NC, normal control.

*** *p* < .001, comparison between NC1 and NC3.

^###^ *p* < .001, comparison between NC1 and AD.

^&&^ *p* < .01.

^&&&^ *p* < .001, comparison between NC1 and MCI.

^§§^ *p* < .01.

^§§§^ *p* < .01, comparison between NC3 and aMCI.

^††^ *p* < .01.

^†††^ *p* < .001, comparison between NC3 and naMCI.

### 3.2 Training of GANCMLAE

After experimental training, the final parameters were improved as follows: the optimisers used by the generator and discriminator were the Adam optimiser, the batch size was 32, and the size of the latent vector was 128. The loss curve recorded by TensorBoard during training is illustrated in Supplementary Figure 1. The training loss could be levelled off quickly using the multiple‐loss alternation training method. After 70,000 batches of training for our deep learning model, the loss of the GAN in the anomaly prediction model converged to zero and the AE tended tend to 0.5 while the performance on validation was consistent with the training data set without overfitting.

### 3.3 Individual generated images and their corresponding residual images

In Supplementary Figure 3, the images on odd‐numbered lines are obtained from T1‐weighted MRI, while the images on even‐numbered lines are their corresponding residual images. Although the T1‐weighted MRI images visually display similar degrees of atrophy degree between NC (1) and NC (2), or between AD (1) and AD (2), they exhibit different individualised atrophy patterns using our GANCMLAE model. For instance, the areas of brain atrophy in patients with AD (1) were larger than those in patients with AD (2), mainly in precuneus, inferior temporal gyrus, median cingulate, and paracingulate gyri, suggesting the heterogeneity in individual level.

### 3.4 Validation of GANCMLAE

#### 3.4.1 Robustness analysis

The SSIM values reached 0.934 ± 0.006 in the validation data set from ADNI, and with the selected model the mean SSIM in the Xuanwu data sets got close to 0.93. The detailed results are depicted in the Supplementary Figure 1, and the SSIM values of the Xuanwu data sets are listed in Supplementary Table 1. All models were constructed for explicit performance comparison, as presented in Table 2. The GANCMLAE model achieves the best performance on SSIM (0.934 ± 0.006) with less latent loss than the other models in the NC2 group, indicating that our model loses the least features and the first step in our task is reasonable. The values of PSNR and MSE are not as good as in our test because the PSNR and MSE are focused on the differences in the pixel level and cannot satisfactorily reflect the subjective feeling of human eyes (Wang et al., 2004; W. Zhou & Bovik, 2002). The DC‐CNN model is not effective for anomaly detection. In addition, the ablation experiments (training the model without L1, L2, or L3, respectively) showed that the SSIM of the ablation experimental model is smaller than that of the GANCMLAE model in both groups, while the PSNR of the ablation experimental model appears to be higher because of the lack of a part of the loss function constraint.

**TABLE 2:** Performance comparison with different baseline models

Models	Train			Test		
	SSIM	PSNR	MSE	SSIM	PSNR	MSE
AE	0.897 ± 0.006	33.87 ± 0.87	0.0008 ± 0.0001	0.889 ± 0.004	34.88 ± 0.38	0.0006 ± 0.0001
VAE	0.886 ± 0.01	35.45 ± 0.13	0.0008 ± 0.0001	0.894 ± 0.003	34.57 ± 0.48	0.0009 ± 0.0001
FAnoGAN	0.804 ± 0.02	25.10 ± 0.14	0.0034 ± 0.0001	0.797 ± 0.006	24.45 ± 0.06	0.0037 ± 0.0001
DC‐CNN	0.879 ± 0.002	**36.99 ± 0.81**	**0.0004 ± 0.0001**	0.884 ± 0.01	**36.45 ± 0.73**	**0.0004 ± 0.0001**
AAE	0.931 ± 0.002	30.75 ± 0.11	0.0016 ± 0.0001	0.927 ± 0.005	30.93 ± 0.12	0.0015 ± 0.0007
GANCMLAE	**0.934 ± 0.006**	31.66 ± 0.07	0.0015 ± 0.0006	**0.929 ± 0.003**	31.04 ± 0.09	0.0014 ± 0.0001
L1 + L2	0.929 ± 0.009	35.49 ± 0.62	0.0006 ± 0.0003	0.929 ± 0.009	35.27 ± 0.78	0.0007 ± 0.0004
L1 + L3	0.889 ± 0.016	33.35 ± 0.10	0.0009 ± 0.0005	0.895 ± 0.014	33.73 ± 0.96	0.0008 ± 0.0003
L2 + L3	0.911 ± 0.015	35.10 ± 0.45	0.0006 ± 0.0003	0.914 ± 0.011	35.21 ± 0.50	0.0006 ± 0.0003

*Note*: The methods are conducted with cross‐validation and their results are given as mean ± standard deviation. The best performing models are highlighted in bold.

Abbreviations: AE, autoencoder; GANCMLAE, generative adversarial networks constrained multiple loss autoencoder; MSE, mean squared error; PSNR, peak signal‐to‐noise ratio; SSIM, structural similarity index measure; VAE, variations encoder.

#### 3.4.2 Dissimilarity assessment

**FIGURE 3:**
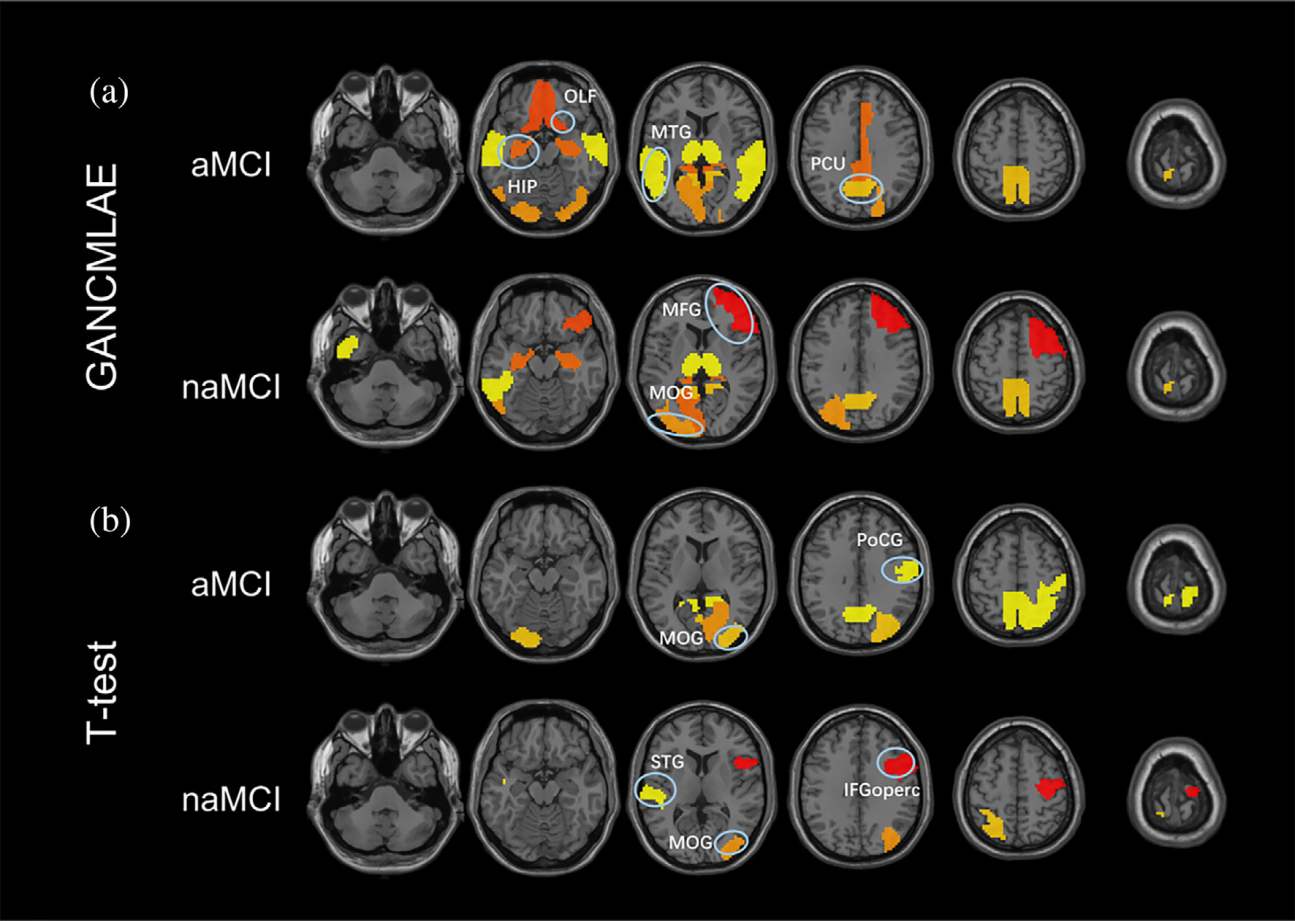
The masks of amnestic mild cognitive impairment (aMCI) and non‐aMCI (naMCI) gained by the generative adversarial network constrained multiple loss autoencoder (GANCMLAE) model and the *t*‐test model. HIP, hippocampus; IFGoperc, inferior frontal gyrus, opercular part; MFG, middle frontal gyrus; MOG, middle occipital gyrus; MTG, middle temporal gyrus; OLF, olfactory cortex; PCU, precuneus; PoCG, postcentral gyrus; STG, superior temporal gyrus

The aMCI and naMCI regional atrophy masks obtained by our method and the t‐test method are displayed in Figure 3. It can be observed that a wider range of brain regions is extracted from the GANCMLAE model compared to the t‐test model. As indicated in Supplementary Table 2, there are 25 characteristic brain areas for the aMCI patients based on the GANCMLAE model, whereas 15 brain areas are extracted using the *t*‐test method. Meanwhile, eight brain regions are shown in the naMCI mask using the GANCMLAE model, while only five regions are found based on the t‐test method. Importantly, the regions with structural abnormalities extracted from the GANCMLAE model differ from those derived from the *t*‐test model; however, those of the former are more consistent with their clinical performance, especially for aMCI patients. Using the GANCMLAE model, the regional atrophy in aMCI patients is focused on the temporal lobe, hippocampus, olfactory cortex, posterior cingulate cortex, and precuneus, whereas the primary atrophy regions are in the occipital lobe, frontal lobe, hippocampus, and precuneus using the *t*‐test method. For naMCI patients, several regions, that are not associated with memory decline, indicating more widespread mild atrophy using the GANCMLAE model than the *t*‐test method.

### 3.5 Application of GANCMLAE

#### 3.5.1 ROC analysis and classification with classical deep learning models

**FIGURE 4:**
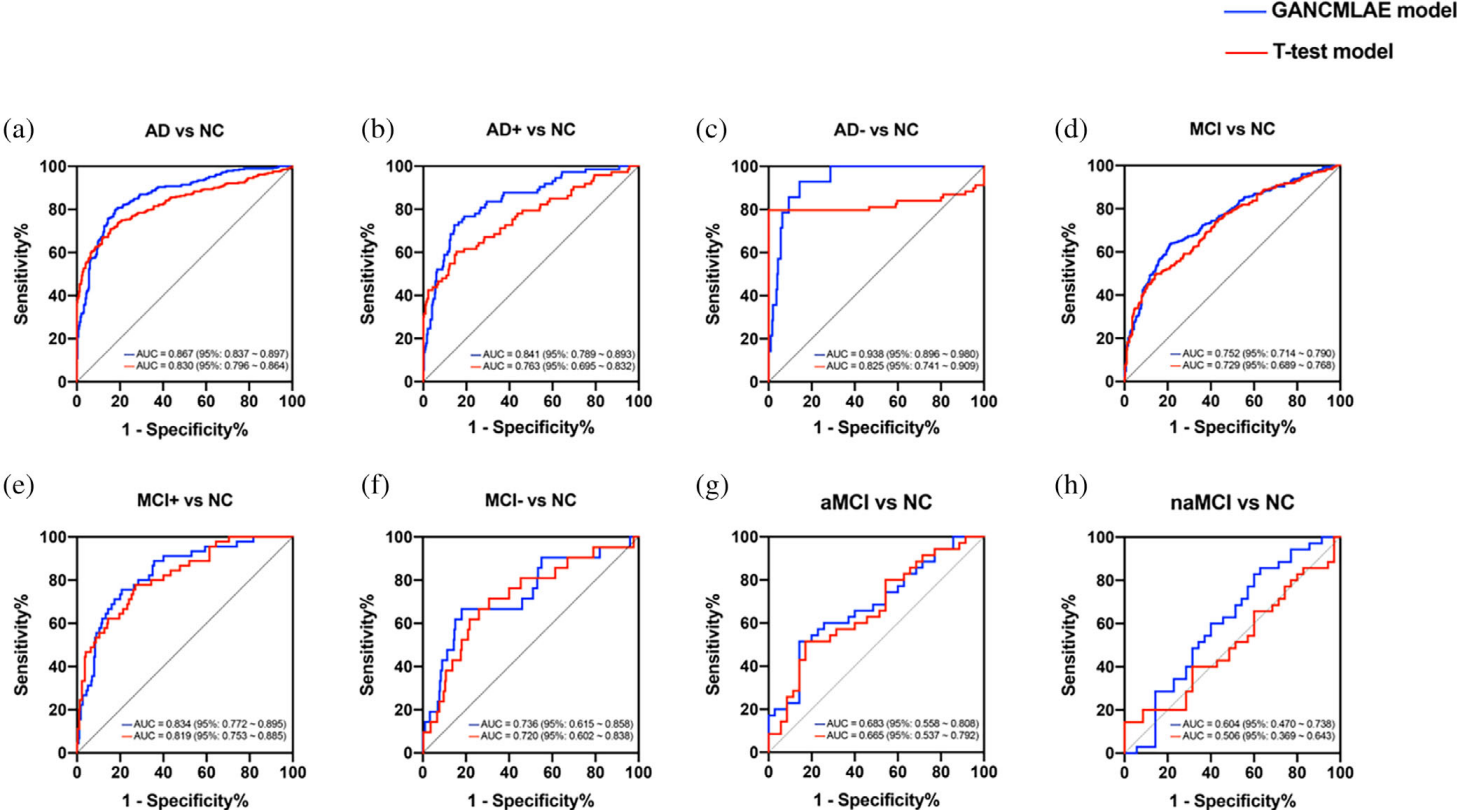
The discriminative power of different models in identifying Alzheimer's disease (AD) and mild cognitive impairment (MCI) from normal control (NC). Red, *t*‐test model; blue, generative adversarial network constrained multiple loss autoencoder (GANCMLAE) model

Using the ROC analysis approach, we first estimated the discriminative power of each of the two models in distinguishing patients with AD from NCs in the ADNI cohort. The individual atrophy patterns from the GANCMLAE model exhibit a relatively good discriminative power, with an AUC of 0.867 (95%: 0.837–0.897) (Figure 4a). The atrophy patterns from the *t*‐test model show a potential discriminative power, with an AUC of 0.830 (95%: 0.796–0.864). In the subgroup analysis for the AD+ and AD− groups, the GANCMLAE model also displays better classification performance than the *t*‐test model (AD+: AUC = 0.841, 95%: 0.789–0.893 vs. AUC = 0.763, 95%: 0.695–0.832, Figure 4b; AD−: AUC = 0.938, 95%: 0.896–0.980 vs. AUC = 0.825, 95%: 0.741–0.909, Figure 4c).

In addition, for MCI patients, the individual atrophy patterns from the GANCMLAE model show a better discriminative power (AUC: 0.752, 95%: 0.714–0.790) than those of *t*‐test model (AUC: 0.729, 95%: 0.689–0.768) (Figure 4d). Moreover, similar findings are observed in the MCI subgroups. The atrophy patterns derived from the GANCMLAE model also have a higher discriminative power in distinguishing MCI+ and MCI− individuals from NC individuals than those of *t*‐test model (MCI+: AUC = 0.834, 95%: 0.772–0.895 vs. AUC = 0.819, 95%: 0.753–0.885, Figure 4e; AD−: AUC = 0.736, 95%: 0.615–0.858 vs. AUC = 0.720, 95%: 0.602–0.838, Figure 4f). We also found that the residual model exhibits a relatively better classification power than the *t*‐test model for discriminating patients with aMCI and naMCI from NCs (Figure 4g,h).

Table 3 lists the classification results of the classical deep learning models. Obviously, with the same model, the residual maps have a better classification potential. In all test and validation data sets, the classification results of the residual images are better than those of the other images. For the classification models discriminating AD from NC individuals, the accuracies of our residual maps are 0.984 ± 0.016 with AlexNet, 0.996 ± 0.008 with ResNet18, 0.984 ± 0.018 with ResNet34, and 0.999 ± 0.004 with LeNet in the test set. In the validation set, the accuracies of our residual maps are 0.981 ± 0.02 with AlexNet, 0.999 ± 0.005 with ResNet18, 0.997 ± 0.005 with ResNet34, and 0.998 ± 0.006 with LeNet. These models exhibit similar classification results in distinguishing MCI from NCs. The accuracies of the residual maps are 0.966 ± 0.047, 0.895 ± 0.07, 0.951 ± 0.028, and 0.993 ± 0.01 with AlexNet, ResNet18, ResNet34, and LeNet, respectively, in the test set. In the validation set, the accuracies of the residual images are 0.954 ± 0.065 with AlexNet, 0.905 ± 0.065 with ResNet18, 0.949 ± 0.039 with ResNet34, and 0.999 ± 0.005 with LeNet. It was proved that the residual maps obtained by our GANCMLAE model possess a better discriminative power for AD and MCI.

**TABLE 3:** Deep learning model classification results and comparison in validation and test sets

		Validation					
AD/NC		Accuracy	Sensitivity	Specificity	AUC	F‐SCORE	MCC
AlexNet	ori	0.916 ± 0.023	0.916 ± 0.029	0.917 ± 0.05	0.917 ± 0.024	0.921 ± 0.021	0.834 ± 0.046
	combineT	0.89 ± 0.039	0.899 ± 0.055	0.878 ± 0.057	0.888 ± 0.038	0.9 ± 0.036	0.779 ± 0.077
	residual	0.981 ± 0.02	0.969 ± 0.037	0.994 ± 0.016	0.982 ± 0.018	0.982 ± 0.019	0.962 ± 0.038
ResNet18	ori	0.882 ± 0.056	0.87 ± 0.113	0.895 ± 0.078	0.883 ± 0.052	0.888 ± 0.062	0.772 ± 0.099
	combineT	0.919 ± 0.012	0.923 ± 0.028	0.916 ± 0.03	0.919 ± 0.011	0.914 ± 0.012	0.839 ± 0.023
	residual	0.999 ± 0.005	0.999 ± 0.008	0.999 ± 0.006	0.999 ± 0.005	0.999 ± 0.005	0.998 ± 0.01
ResNet34	ori	0.898 ± 0.04	0.904 ± 0.082	0.891 ± 0.084	0.898 ± 0.039	0.907 ± 0.04	0.802 ± 0.071
	combineT	0.92 ± 0.03	0.921 ± 0.044	0.918 ± 0.046	0.919 ± 0.03	0.925 ± 0.029	0.84 ± 0.059
	residual	0.997 ± 0.005	0.998 ± 0.005	0.995 ± 0.009	0.997 ± 0.005	0.997 ± 0.004	0.994 ± 0.009
LeNet	ori	0.936 ± 0.019	0.929 ± 0.034	0.944 ± 0.001	0.936 ± 0.017	0.94 ± 0.018	0.871 ± 0.036
	combineT	0.91 ± 0.03	0.926 ± 0.05	0.89 ± 0.075	0.908 ± 0.033	0.92 ± 0.027	0.821 ± 0.058
	residual	0.998 ± 0.006	0.996 ± 0.011	0.999 ± 0.001	0.998 ± 0.006	0.998 ± 0.006	0.996 ± 0.011

		Test					
AD/NC		Accuracy	Sensitivity	Specificity	AUC	F‐SCORE	MCC
AlexNet	ori	0.888 ± 0.008	0.864 ± 0.018	0.92 ± 0.028	0.892 ± 0.01	0.899 ± 0.007	0.776 ± 0.018
	combineT	0.901 ± 0.014	0.934 ± 0.027	0.873 ± 0.029	0.903 ± 0.013	0.896 ± 0.014	0.805 ± 0.027
	residual	0.984 ± 0.016	0.979 ± 0.023	0.99 ± 0.015	0.985 ± 0.016	0.985 ± 0.015	0.968 ± 0.033
ResNet18	ori	0.89 ± 0.021	0.889 ± 0.042	0.892 ± 0.055	0.89 ± 0.021	0.888 ± 0.02	0.783 ± 0.042
	combineT	0.921 ± 0.014	0.893 ± 0.018	0.944 ± 0.023	0.919 ± 0.014	0.912 ± 0.015	0.841 ± 0.029
	residual	0.996 ± 0.008	0.996 ± 0.012	0.996 ± 0.01	0.996 ± 0.008	0.996 ± 0.008	0.992 ± 0.015
ResNet34	ori	0.907 ± 0.021	0.925 ± 0.046	0.891 ± 0.058	0.908 ± 0.019	0.901 ± 0.02	0.818 ± 0.036
	combineT	0.922 ± 0.015	0.913 ± 0.018	0.931 ± 0.023	0.922 ± 0.015	0.918 ± 0.016	0.845 ± 0.03
	residual	0.984 ± 0.018	0.996 ± 0.01	0.968 ± 0.044	0.982 ± 0.021	0.986 ± 0.016	0.968 ± 0.036
LeNet	ori	0.915 ± 0.011	0.978 ± 0.031	0.842 ± 0.012	0.91 ± 0.009	0.925 ± 0.011	0.834 ± 0.027
	combineT	0.924 ± 0.015	0.957 ± 0.006	0.892 ± 0.032	0.924 ± 0.014	0.925 ± 0.013	0.85 ± 0.027
	residual	0.999 ± 0.004	0.999 ± 0.007	0.999 ± 0.001	0.999 ± 0.003	0.999 ± 0.003	0.999 ± 0.007

		Validation					
MCI/NC		Accuracy	Sensitivity	Specificity	AUC	F‐SCORE	MCC
AlexNet	ori	0.779 ± 0.05	0.767 ± 0.073	0.791 ± 0.121	0.779 ± 0.052	0.781 ± 0.044	0.564 ± 0.097
	combineT	0.818 ± 0.032	0.829 ± 0.071	0.805 ± 0.067	0.817 ± 0.032	0.823 ± 0.036	0.639 ± 0.062
	residual	0.954 ± 0.065	0.902 ± 0.14	0.999 ± 0.004	0.951 ± 0.07	0.942 ± 0.087	0.915 ± 0.117
ResNet18	ori	0.773 ± 0.024	0.736 ± 0.069	0.811 ± 0.091	0.774 ± 0.025	0.768 ± 0.025	0.554 ± 0.048
	combineT	0.79 ± 0.039	0.777 ± 0.063	0.804 ± 0.063	0.79 ± 0.039	0.791 ± 0.041	0.583 ± 0.078
	residual	0.905 ± 0.065	0.975 ± 0.034	0.844 ± 0.126	0.91 ± 0.062	0.909 ± 0.058	0.826 ± 0.115
ResNet34	ori	0.783 ± 0.025	0.758 ± 0.041	0.811 ± 0.061	0.784 ± 0.025	0.782 ± 0.023	0.57 ± 0.05
	combineT	0.793 ± 0.024	0.776 ± 0.051	0.81 ± 0.044	0.793 ± 0.024	0.793 ± 0.028	0.588 ± 0.048
	residual	0.949 ± 0.039	0.957 ± 0.066	0.939 ± 0.076	0.948 ± 0.04	0.946 ± 0.043	0.903 ± 0.073
LeNet	ori	0.784 ± 0.778	0.703 ± 0.706	0.859 ± 0.846	0.781 ± 0.776	0.758 ± 0.754	0.571 ± 0.559
	combineT	0.866 ± 0.037	0.855 ± 0.051	0.878 ± 0.037	0.866 ± 0.036	0.868 ± 0.038	0.733 ± 0.072
	residual	0.999 ± 0.005	0.997 ± 0.012	0.999 ± 0.001	0.998 ± 0.006	0.998 ± 0.006	0.997 ± 0.011

		Test					
MCI/NC		Accuracy	Sensitivity	Specificity	AUC	F‐SCORE	MCC
AlexNet	ori	0.764 ± 0.027	0.775 ± 0.067	0.754 ± 0.089	0.765 ± 0.026	0.761 ± 0.024	0.535 ± 0.049
	combineT	0.766 ± 0.014	0.778 ± 0.063	0.755 ± 0.048	0.767 ± 0.015	0.762 ± 0.023	0.536 ± 0.029
	residual	0.966 ± 0.047	0.929 ± 0.113	0.993 ± 0.013	0.961 ± 0.056	0.955 ± 0.066	0.933 ± 0.09
ResNet18	ori	0.772 ± 0.02	0.746 ± 0.06	0.795 ± 0.057	0.771 ± 0.02	0.759 ± 0.026	0.545 ± 0.041
	combineT	0.768 ± 0.018	0.757 ± 0.062	0.779 ± 0.05	0.768 ± 0.019	0.759 ± 0.026	0.539 ± 0.036
	residual	0.895 ± 0.07	0.95 ± 0.043	0.854 ± 0.12	0.902 ± 0.064	0.888 ± 0.069	0.802 ± 0.128
ResNet34	ori	0.773 ± 0.025	0.743 ± 0.059	0.8 ± 0.044	0.772 ± 0.025	0.759 ± 0.032	0.546 ± 0.049
	combineT	0.77 ± 0.031	0.757 ± 0.044	0.782 ± 0.057	0.769 ± 0.03	0.761 ± 0.03	0.541 ± 0.062
	residual	0.951 ± 0.028	0.917 ± 0.082	0.976 ± 0.031	0.947 ± 0.034	0.939 ± 0.037	0.903 ± 0.053
LeNet	ori	0.797 ± 0.794	0.677 ± 0.673	0.884 ± 0.883	0.78 ± 0.778	0.737 ± 0.734	0.581 ± 0.575
	combineT	0.825 ± 0.029	0.799 ± 0.03	0.849 ± 0.054	0.824 ± 0.028	0.815 ± 0.027	0.65 ± 0.058
	residual	0.993 ± 0.01	0.996 ± 0.014	0.991 ± 0.016	0.993 ± 0.01	0.993 ± 0.01	0.986 ± 0.019

Abbreviations: AD, Alzheimer's disease; AUC, area under curve; combineT, original images supervised by *t*‐test masks; MCC, Matthews correlation coefficient; MCI: mild cognitive impairment; NC, normal control; ori, original.

#### 3.5.2 Correlation analysis

**FIGURE 5:**
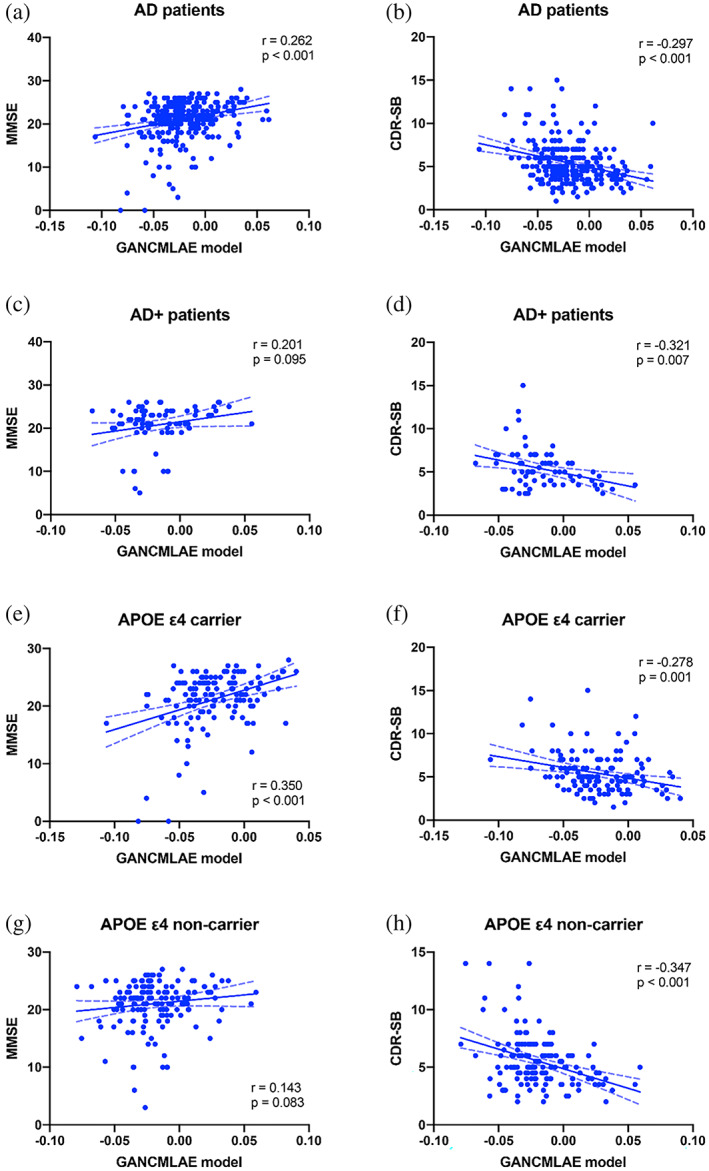
The associations of different models with cognitive performance in Alzheimer's disease (AD)

Partial correlation analysis was performed to evaluate the correlation between the different models and cognitive performance, using age, sex, and years of education as covariates. As shown in Figure 5, a significant positive correlation exists between the individual atrophy patterns from the GANCMLAE model and MMSE (*r* = .262, *p* < .001) (Figure 5a), while there is no correlation between the atrophy patterns from the *t*‐test model and MMSE (*r* = .066, *p* = .262). For the severity of cognition, a significantly negative correlation between the CDR‐SB and the GANCMLAE model (*r* = −.297, *p* < .001) (Figure 5b) is observed. In contrast, no significant correlation between the *t*‐test model and CDR‐SB (*r* = −.085, *p* = .152) exists, suggesting that the individual atrophy patterns from the GANCMLAE model can serve as good indicators for reflecting the cognitive decline.

In the subgroup analysis, the AD+ group shows a significant negative correlation between the CDR‐SB and individual atrophy patterns from the GANCMLAE model (*r* = −.321, *p* = .007) (Figure 5d), whereas no statistically significant correlation is observed between MMSE and the GANCMLAE model (*r* = .201, *p* = .095) (Figure 5c). There is no significant correlation between the cognitive performance (MMSE and CDR‐SB) and GANCMLAE model for the AD− group. Furthermore, no significant correlation between the *t*‐test model and MMSE and CDR‐SB in both the AD+ and AD− groups is found. However, in the MCI+ subgroup, significant correlations between MMSE and both models exist. Additionally, there is a significant negative correlation between CDR‐SB and the *t*‐test model in the MCI− subgroup (Supplementary Figure 4).

For APOE ε4 carriers, significant correlations between the cognitive performance (MMSE and CDR‐SB) and individual atrophy patterns from the GANCMLAE model are observed (MMSE: *r* = .350, *p* < .001, Figure 5e; CDR‐SB: *r* = −.278, *p* = .001, Figure 5f). For APOE ε4 noncarriers, the GANCMLAE model is negatively associated with CDR‐SB (*r* = −.347, *p* < .001, Figure 5h), but not with MMSE (*r* = .143, *p* = .083, Figure 5g). Using the *t*‐test model, only the negative association of the atrophy patterns from the *t*‐test model and CDR‐SB is observed for the APOE ε4 noncarriers (*r* = −.165, *p* = .045). Furthermore, a significant interaction effect is found between the APOE genotype and GANCMLAE model in the MMSE with AD patients (*F*_(1, 281)_ = 5.089, *p* = .025). However, no significant interaction effect is observed between the APOE genotype and GANCMLAE model in CDR‐SB (*F*_(1, 281)_ = 0.790, *p* = .375), or between the APOE genotype and the *t*‐test model in MMSE (*F*_(1, 281)_ = 0.257, *p* = .613) and CDR‐SB (*F*_(1, 281)_ = 2.212, *p* = .138).

#### 3.5.3 Predictive effect on the conversion risk of MCI to dementia

**FIGURE 6:**
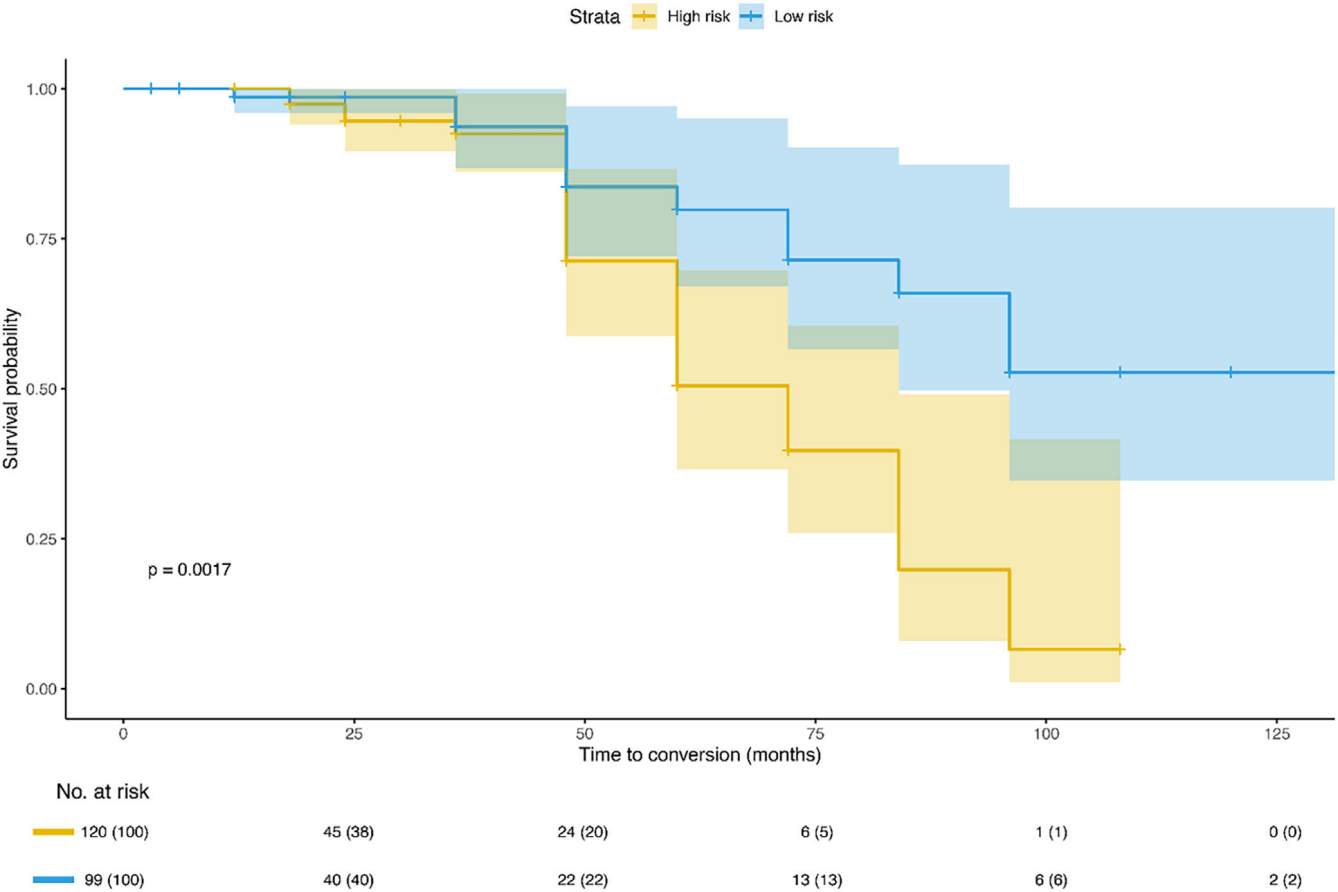
The predictive effect of the residual model on the conversion risk of mild cognitive impairment (MCI) to dementia

In the survival analysis of MCI patients from the ADNI cohort, higher residual scores (standardised residual score >0, red line) indicate a better predictive effect for developing a clinical diagnosis of dementia than lower residual scores (standardised residual score <0, blue line) (HR: 2.493, 95% CI: 1.349–4.605, *p* = .0017, Figure 6).

## 4 DISCUSSION

In the present study, we developed the novel GANCMLAE model, and cross‐validation was conducted in both the ADNI and Xuanwu cohorts. The results showed that the GANCMLAE model had a higher potential for detecting individual atrophy patterns for AD and MCI than the group‐level *t*‐test model. Moreover, the associations of individual atrophy patterns from the GANCMLAE model with the global cognitive function and cognitive severity were observed. Finally, the individual atrophy patterns further demonstrated the predictive effect of conversion to dementia for MCI patients during the follow‐up.

The GANCMLAE, with its outstanding learning ability and discernibility, utilises a GAN to constrain the process of data codec in AE, leading to more accurate mapped images (Cao et al., 2021; Yu et al., 2020). In a previous study involving anomaly detection or generation, the loss was only used as a score to distinguish the target, without guaranteeing the feature recovery of the generated image. The added loss L3 of the latent vector can satisfactorily preserve “normal” information and make the total loss accurately indicate abnormality in the phase of atrophy detection. The alternating training makes the image reconstruction procedure more stable, and the total loss converges faster. Importantly, the GANCMLAE model provides insight into individual structural alterations relative to the *t*‐test model, which focuses on group‐level differences. In our study, although the GANCMLAE model sacrificed some pixel accuracy to maintain more features and achieve a balanced reconstruction performance, the comparison of four different models (LeNet, AlexNet, ResNet18, and ResNet34 models) showed the outstanding learning ability of the GANCMLAE in the reconstruction task, which is fundamental for the subsequent detection of individual atrophy.

In this study, the range of regional brain atrophy extracted from the GANCMLAE model of aMCI and naMCI seemed to be wider than the atrophy patterns derived from the *t*‐test model, indicating that the deep learning algorithm may have the potential to better reflect individual heterogeneity. Additionally, the characteristic brain atrophy patterns based on the residual model of aMCI patients were more consistent with the clinical symptoms. To the best of our knowledge, aMCI, which is the amnestic subtype of MCI and constitutes a prodromal stage of AD, has a high risk of progression to AD (Gauthier et al., 2006). Using structural MRI, researchers have reported a significant GM volume reduction in the medial temporal cortices (e.g., hippocampus) in aMCI patients (Lim et al., 2012; Sheng et al., 2020). Notably, hippocampal atrophy is strongly correlated with memory loss. Moreover, it has been reported that in addition to the medial temporal lobe, patients with AD present significant posterior cingulate gyrus and precuneus atrophy, especially in early onset individuals (Koedam et al., 2011; Persson et al., 2017). In our previous study, we confirmed significant regional brain atrophy in the medial temporal lobe and posterior cortex atrophy in aMCI patients (Sheng et al., 2020). For naMCI patients, more brain regions that are not associated with the memory function, such as the parietal lobe, occipital lobe, and calcarine, were also found based on the residual model than on the *t*‐test model, suggesting the potential of deep learning methods for precision diagnosis.

The individual atrophy patterns from the GANCMLAE exhibited a relatively better discriminative power in identifying the targeted population, including patients with AD and MCI than those of the conventional methods. Moreover, the GANCMLAE model was associated with the clinical cognitive function in AD, and the individual atrophy patterns from GANCMLAE could serve as a topographical biomarker for indicating MCI progression.

In the clinical applications, the individual atrophy patterns from GANCMLAE exhibited a relatively better discriminative power in identifying the targeted population, including patients with AD and MCI, than those of the *t*‐test model, which verified the diagnostic potential of our deep learning model. In addition, the GANCMLAE model was associated with the clinical cognitive function in AD, especially in individuals with Aβ‐positive AD. However, there was no significant correlation between the *t*‐test scores and cognitive performance, suggesting that the residual index may be a better indicator for reflecting cognitive decline in AD patients. It is noteworthy that in the MCI subgroup analysis, the *t*‐test model appears to be more relevant to the patients' cognitive impairment than individual atrophy patterns detected in the GANCMLAE model. We infer this potential mechanism may be associated with the clinical heterogeneity of MCI patients. In our study, we confirmed that regional brain atrophy patterns are significantly different between aMCI and naMCI. Furthermore, we investigated the ability of residual maps to predict the conversion risk of MCI individuals to dementia. Many previous studies have reported that regional brain atrophy may represent a predictor of cognitive decline from MCI to dementia (Dai & He, 2014; Li et al., 2020; Wei et al., 2016). In this study, we also confirmed that the individual atrophy patterns from GANCMLAE can serve as a topographical biomarker for indicating the progression of MCI. In summary, the GANCMLAE model based on a deep learning algorithm may provide a potential avenue for achieving precise individualised prediction.

It should be noted that this study has some limitations. First, a certain disparity remains between the generated image and the original input in terms of resolution, and the performance of the model needs to be further improved. This disparity may be attributed to the collection of structural MRI data from different machines in the two cohorts. In the future, strategies should be implemented to ensure consistency in multi‐centre, cross‐machine data collection. Second, the underlying physiological mechanisms of residual maps for AD and MCI were not elucidated in the present study. Future studies should investigate the correlation between residual images and human molecular pathways, such as neurotransmitters and AD‐related pathological changes. Furthermore, in our study, we only used 2D images for the reconstruction of models because the longer processing time and higher memory requirement when utilising 3D images. In the future, one possible extension could be the use of 3D images instead of 2D for model reconstruction if the computing power is improved. Finally, given that the sample size in our study is not large, the *p* value is relatively large, and *p* < .05 may not be sufficient for many comparisons. Although we used a cross‐validation method to validate our findings, multiple comparisons are still issues that need to be considered. In the future, multi‐centre studies with a larger sample size are essential to provide more accurate evidence.

## 5 CONCLUSION

Our study developed the GANCMLAE model for the detection of individual atrophy patterns based on structural MRI data. Experiments on two independent cohorts of participants showed that the residual maps from the GANCMLAE model can serve as an effective tool for achieving precise individualised atrophy detection and have potential clinical applications.

## AUTHOR CONTRIBUTIONS

Rong Shi: Writing‐original draft, methodology, validation. Can Sheng: Writing‐original draft, methodology. Shichen Jin: Resources, software. Qi Zhang: Resources, methodology. Shuoyan Zhang: Formal analysis, validation. Liang Zhang: Visualisation. Changchang Ding: Visualisation. Luyao Wang: Software. Lei Wang: Writing‐review and editing. Ying Han: Supervision, funding. Jiehui Jiang: Investigation, project administration.

## CONFLICT OF INTEREST

The authors declare that they have no known competing financial interests or personal relationships that could have appeared to influence the work reported in this article.

## Supporting information


**APPENDIX S1** Supporting information

## Data Availability

This study used data from two cohorts: Alzheimer's Disease Neuroimaging Initiative (ADNI) and Xuanwu Hospital of Capital Medical University. The data of the ADNI cohort were originally from the online repository of the ADNI database (http://adni.loni.usc.edu/), which is easily available for the research public. The data from Xuanwu cohort and generated during processing and analysis are available from the corresponding author upon reasonable request. And the underlying code is updated at Zen‐keeper/cAAE‐new (github.com).
