# Supplementary material for: Generative adversarial network constrained multiple loss autoencoder: A deep learning‐based individual atrophy detection for Alzheimer's disease and mild cognitive impairment
Source: Hum Brain Mapp. 2022 Nov 17;44(3):1129–46. doi: 10.1002/hbm.26146 (PMC9875916; doi:10.1002/hbm.26146)
Supplement: Supplementary file 1 — APPENDIX S1 Supporting information [file HBM-44-1129-s001.pdf]

Supplementary Appendix

Supplementary Figures:

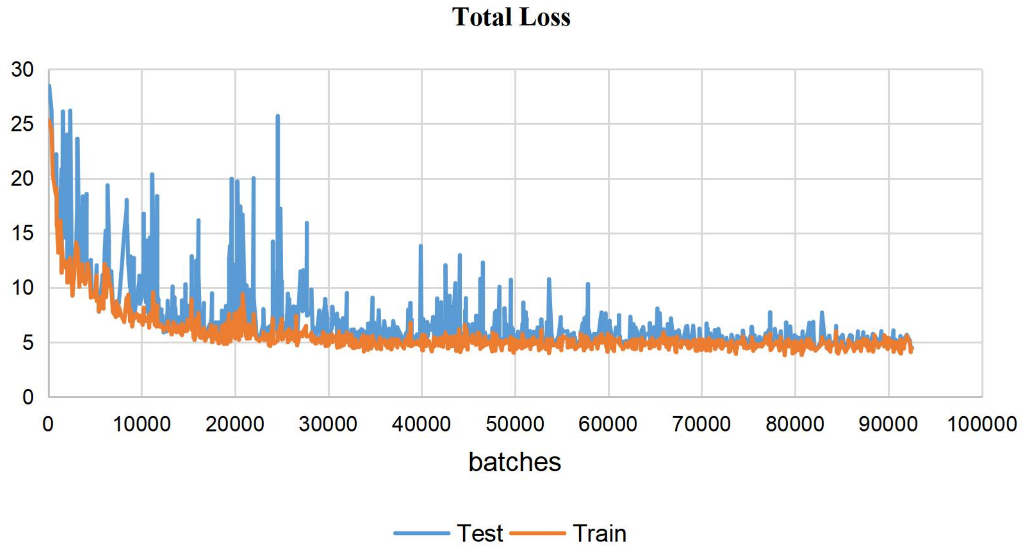

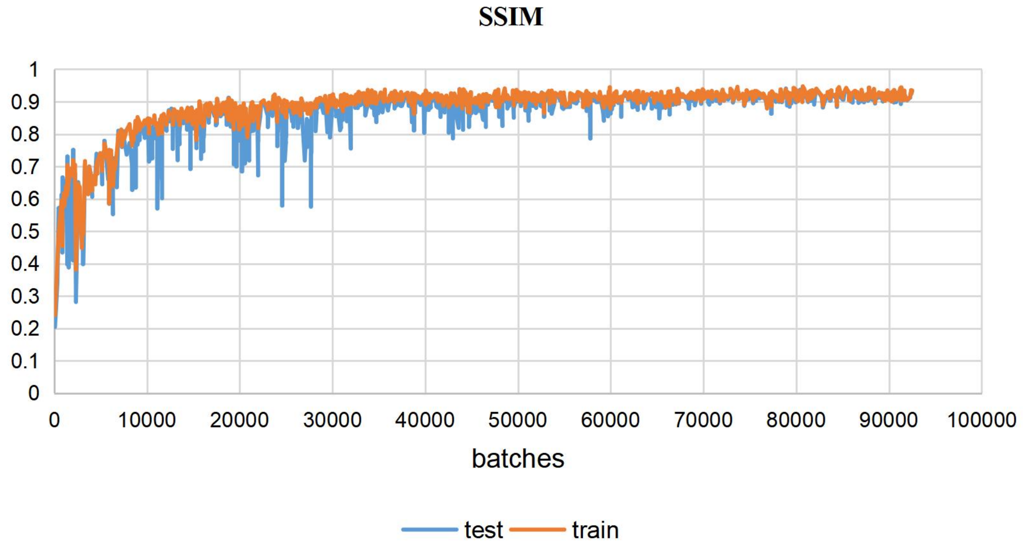

Supplementary Figure 1 One of the training procedures of GANCMLAE with L3 loss. After 70000 batches if training the loss is almost stable.

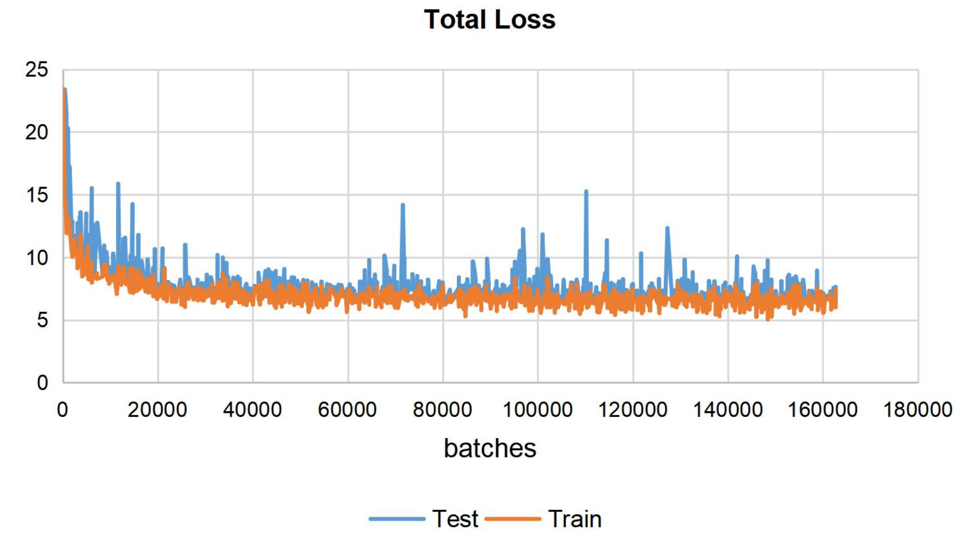

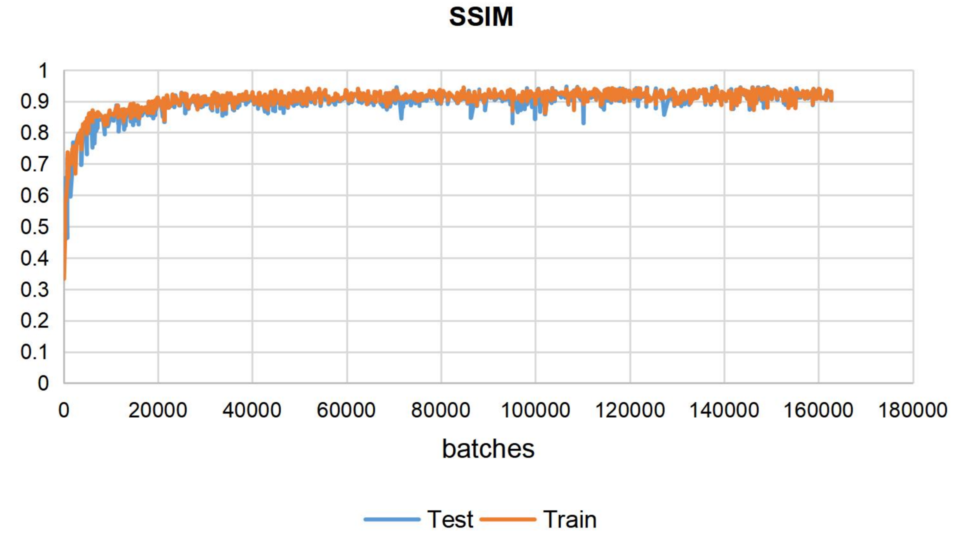

Supplementary Figure 2 One of the training procedures without L3 loss. The loss is worse and oscillated greater than the above loss.

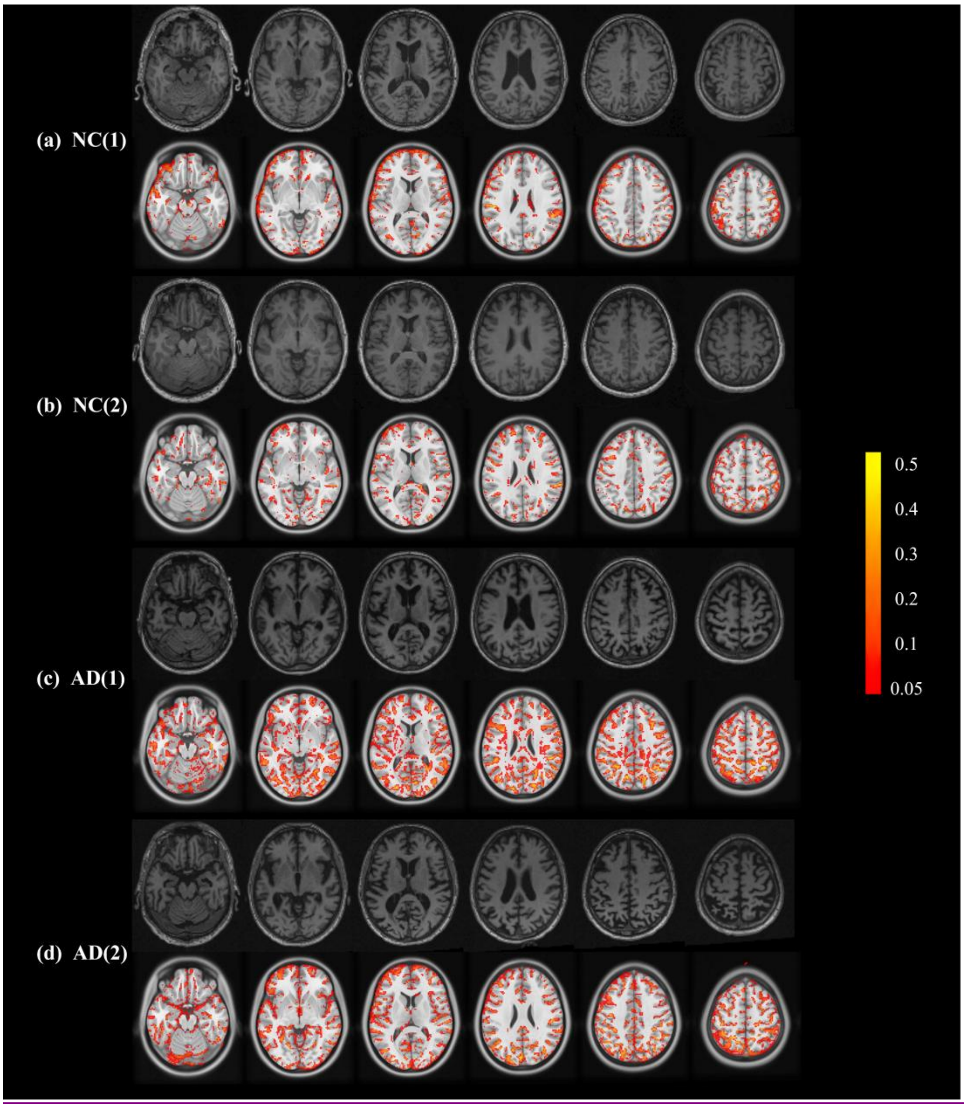

Supplementary Figure 3 Individual generated images and their corresponding residual images for NC and AD.

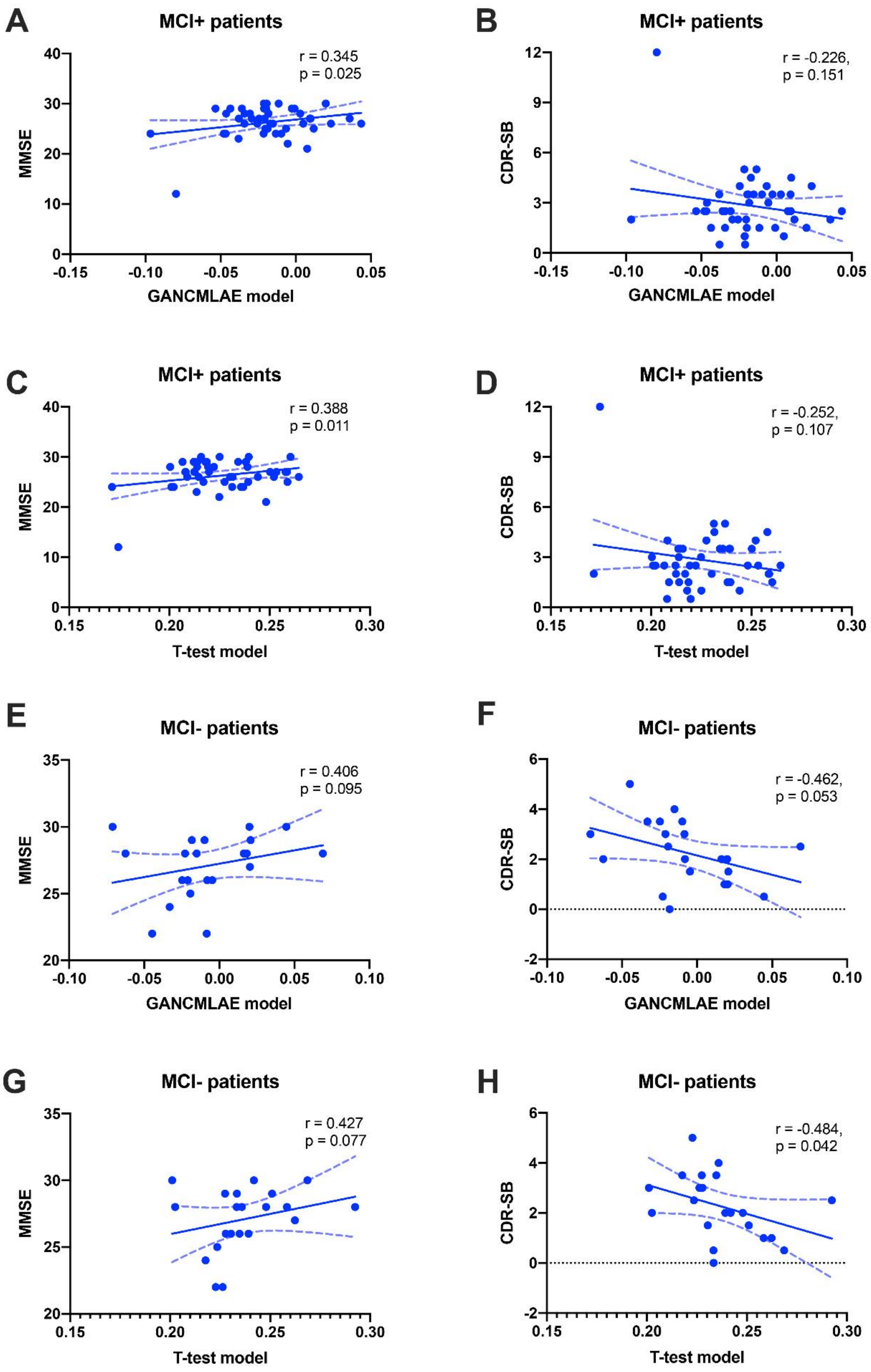

Supplementary Figure 4 The association of the two models with MMSE and CDR-SB in the MCI subgroups.

Supplementary Table1 SSIM of NCs in Xuanwu cohort (NC3 group)

subjects	mean SSIM	subjects	mean SSIM
sub001	0.931624	sub054	0.932838
sub002	0.931481	sub055	0.930494
sub003	0.932873	sub056	0.928279
sub004	0.933612	sub057	0.931777
sub005	0.926053	sub058	0.936816
sub006	0.931054	sub059	0.936124
sub007	0.934149	sub060	0.936343
sub008	0.934124	sub061	0.932032
sub009	0.932226	sub062	0.931678
sub010	0.929703	sub063	0.9349
sub011	0.937052	sub064	0.938053
sub012	0.933672	sub065	0.929941
sub013	0.936543	sub066	0.937585
sub014	0.933489	sub067	0.924162
sub015	0.940298	sub068	0.934372
sub016	0.935456	sub069	0.935235
sub017	0.936207	sub070	0.934333
sub018	0.929376	sub071	0.935294
sub019	0.932865	sub072	0.934234
sub020	0.924912	sub073	0.937425
sub021	0.934792	sub074	0.93106
sub022	0.9334	sub075	0.935755
sub023	0.934979	sub076	0.935057
sub024	0.930703	sub077	0.936376
sub025	0.937122	sub078	0.93378
sub026	0.935989	sub079	0.937833
sub027	0.93369	sub080	0.939946
sub028	0.930331	sub081	0.94212
sub029	0.925796	sub082	0.927253
sub030	0.933391	sub083	0.929888
sub031	0.934427	sub084	0.938013
sub032	0.935061	sub085	0.930511
sub033	0.917336	sub086	0.934412
sub034	0.9163	sub087	0.938577
sub035	0.929218	sub088	0.937138
sub036	0.931947	sub089	0.934694
sub037	0.932933	sub090	0.93507
sub038	0.934362	sub091	0.934974
sub039	0.935608	sub092	0.935447
sub040	0.937247	sub093	0.934863
sub041	0.929544	sub094	0.932697

sub042	0.934104	sub095	0.935275
sub043	0.934152	sub096	0.931209
sub044	0.934055	sub097	0.934736
sub045	0.934655	sub098	0.939306
sub046	0.935775	sub099	0.929284
sub047	0.938808	sub100	0.938543
sub048	0.930787	sub101	0.93631
sub049	0.928477	sub102	0.934384
sub050	0.927781	sub103	0.932566
sub051	0.926033	sub104	0.936483
sub052	0.930109	sub105	0.92689
Sub053	0.929246		

Supplementary Table2 Brain regional atrophy in aMCI and naMCI based on the residual model and t-test model

**GANCMLAE (aMCI)**		**T-test (aMCI)**	
Cluster location (AAL template)	Cluster size	Cluster location (AAL template)	Cluster size
Thalamus_R, Pallidum_R, Putamen_R, Rectus_R, Caudate_R, Frontal_Sup_Orb_R	1003	Cerebelum_3_R Lingual_R	49
Temporal_Pole_Mid_L, Temporal_Inf_L	63	ParaHippocampal_R	12
Temporal_Inf_R, Temporal_Mid_R	251	Hippocampus_L	29
Temporal_Sup_L, Temporal_Mid_L, Heschl_L	174	Hippocampus_R	38
Lingual_R, Cerebelum_6_R, Fusiform_R, Precuneus_R, Calcarine_R, Cerebelum_8_R, Cerebelum_8_L, Thalamus_L, Occipital_Mid_L, Temporal_Mid_L, Fusiform_L, Occipital_Mid_R, Cuneus_R, Temporal_Inf_L, Cerebelum_Crus2_R, Occipital_Inf_R, ParaHippocampal_R, Occipital_Sup_L, Cingulum_Post_L, Occipital_Sup_R, Pallidum_L, Cingulum_Post_R, Calcarine_L, Cerebelum_Crus1_R, Cerebelum_9_L, Cerebelum_4_5_R, Putamen_L, Cerebelum_9_R, Hippocampus_L, Cerebelum_6_L, ParaHippocampal_L, Occipital_Inf_L, Temporal_Mid_R, Frontal_Sup_Orb_L, Temporal_Inf_R, Cingulum_Mid_R, SupraMarginal_L	10759	Frontal_Mid_R, Frontal_Inf_Tri_R, Frontal_Sup_R, Frontal_Mid_Orb_R, Temporal_Mid_R, Frontal_Sup_Medial_R, Temporal_Sup_R, Frontal_Inf_Orb_R, Frontal_Sup_Orb_R, Postcentral_R, Precentral_R, Rectus_R, Temporal_Inf_R, Frontal_Med_Orb_R, Parietal_Sup_R, Frontal_Inf_Oper_R, Parietal_Inf_R, Rolandic_Oper_R, Cingulum_Ant_R, Temporal_Pole_Sup_R, Temporal_Pole_Mid_R	5003

Hippocampus_R, ParaHippocampal_R	232	Occipital_Mid_L, Angular_L, Occipital_Inf_L, Occipital_Sup_L, Temporal_Mid_L, Parietal_Sup_L, Parietal_Inf_L, Lingual_L, Precuneus_L, Cuneus_L, SupraMarginal_L, Hippocampus_L, Fusiform_L, Temporal_Inf_L	5523
Frontal_Mid_Orb_L, Frontal_Mid_L, Frontal_Inf_Orb_L	136	Hippocampus_R, ParaHippocampal_R	67
SupraMarginal_R, Temporal_Sup_R, Heschl_R	122	Calcarine_R	49
Frontal_Inf_Oper_L, Frontal_Inf_Tri_L	79	Cuneus_R, Occipital_Sup_R, Calcarine_R	82
Angular_R, Occipital_Mid_R, Parietal_Sup_R	188	Temporal_Sup_L	41
Parietal_Sup_R, Postcentral_R, Precentral_R, Frontal_Mid_R, Parietal_Inf_R	628	Thalamus_R, Caudate_R	59
Parietal_Sup_L, Postcentral_L, Precentral_L	354	Precuneus_R	51

**GANCMLAE (naMCI)**		**T-test (naMCI)**	
Cluster location (AAL template)	Cluster size	Cluster location (AAL template)	Cluster size
Temporal_Pole_Mid_R, Temporal_Inf_R	106	Temporal_Sup_L	63
Occipital_Mid_R, Occipital_Inf_R, Occipital_Sup_R, Temporal_Mid_R	281	Precentral_R, Postcentral_R, Rolandic_Oper_R	109

Parietal_Sup_R, Frontal_Mid_R, Postcentral_R, Precentral_R, Frontal_Inf_Tri_R, Parietal_Inf_R, Frontal_Sup_R, Frontal_Inf_Orb_R, Angular_R, Temporal_Pole_Sup_R, Frontal_Inf_Oper_R	2052	Frontal_Mid_R, Frontal_Inf_Tri_R	82
Frontal_Mid_R	59	Occipital_Sup_R	67
Lingual_L, Precuneus_L	88	Parietal_Sup_R, Angular_R	58
Frontal_Mid_L, Frontal_Sup_L	238	Parietal_Sup_L	55
Frontal_Inf_Oper_L	47	Frontal_Sup_R	60
Postcentral_L	90		
Parietal_Sup_L, Postcentral_L, Precuneus_L, Precentral_L	345		
Supp_Motor_Area_R	76		
